# Neural stem cell transplantation in patients with progressive multiple sclerosis: an open-label, phase 1 study

**DOI:** 10.1038/s41591-022-02097-3

**Published:** 2023-01-09

**Authors:** Angela Genchi, Elena Brambilla, Francesca Sangalli, Marta Radaelli, Marco Bacigaluppi, Roberto Furlan, Annapaola Andolfo, Denise Drago, Cinzia Magagnotti, Giulia Maria Scotti, Raffaella Greco, Paolo Vezzulli, Linda Ottoboni, Marco Bonopane, Daniela Capilupo, Francesca Ruffini, Daniela Belotti, Benedetta Cabiati, Stefania Cesana, Giada Matera, Letizia Leocani, Vittorio Martinelli, Lucia Moiola, Luca Vago, Paola Panina-Bordignon, Andrea Falini, Fabio Ciceri, Anna Uglietti, Maria Pia Sormani, Giancarlo Comi, Mario Alberto Battaglia, Maria A. Rocca, Loredana Storelli, Elisabetta Pagani, Giuseppe Gaipa, Gianvito Martino

**Affiliations:** 1 Neuroimmunology Unit, Institute of Experimental Neurology, IRCCS San Raffaele Scientific Institute, Milan, Italy; 2 Department of Neurology, IRCCS San Raffaele Scientific Institute, Milan, Italy; 3 University Vita-Salute San Raffaele, Milan, Italy; 4 Clinical Neuroimmunology Unit, Institute of Experimental Neurology, IRCCS San Raffaele Scientific Institute, Milan, Italy; 5 ProMeFa, Proteomics and Metabolomics Facility, Center for Omics Sciences (COSR), IRCCS San Raffaele Scientific Institute, Milan, Italy; 6 Center for Omics Sciences (COSR), IRCCS San Raffaele Scientific Institute, Milan, Italy; 7 Haematology and Bone Marrow Transplantation Unit, IRCCS San Raffaele Scientific Institute, Milan, Italy; 8 Department of Neuroradiology and CERMAC, IRCCS San Raffaele Scientific Institute, Milan, Italy; 9 Clinical Trial Center, IRCCS San Raffaele Scientific Institute, Milan, Italy; 10 M. Tettamanti Research Center, Pediatric Clinic University of Milano-Bicocca, San Gerardo Hospital, Monza, Italy; 11 Laboratorio di Terapia Cellulare e Genica Stefano Verri, ASST-Monza, Ospedale San Gerardo, Monza, Italy; 12 Department of Gynaecology, IRCCS Ca’ Granda Ospedale Maggiore Policlinico, Milan, Italy; 13 Biostatistics Unit, Department of Health Sciences (DISSAL), University of Genoa, Genoa, Italy; 14 Italian Multiple Sclerosis Foundation, Genoa, Italy; 15 Neuroimaging Research Unit, Division of Neuroscience, IRCCS San Raffaele Scientific Institute, Milan, Italy

**Keywords:** Multiple sclerosis, Neural stem cells, Regeneration and repair in the nervous system

## Abstract

Innovative pro-regenerative treatment strategies for progressive multiple sclerosis (PMS), combining neuroprotection and immunomodulation, represent an unmet need. Neural precursor cells (NPCs) transplanted in animal models of multiple sclerosis have shown preclinical efficacy by promoting neuroprotection and remyelination by releasing molecules sustaining trophic support and neural plasticity. Here we present the results of STEMS, a prospective, therapeutic exploratory, non-randomized, open-label, single-dose-finding phase 1 clinical trial (NCT03269071, EudraCT 2016-002020-86), performed at San Raffaele Hospital in Milan, Italy, evaluating the feasibility, safety and tolerability of intrathecally transplanted human fetal NPCs (*hf*NPCs) in 12 patients with PMS (with evidence of disease progression, Expanded Disability Status Scale ≥6.5, age 18–55 years, disease duration 2–20 years, without any alternative approved therapy). The safety primary outcome was reached, with no severe adverse reactions related to *hf*NPCs at 2-year follow-up, clearly demonstrating that *hf*NPC therapy in PMS is feasible, safe and tolerable. Exploratory secondary analyses showed a lower rate of brain atrophy in patients receiving the highest dosage of *hf*NPCs and increased cerebrospinal fluid levels of anti-inflammatory and neuroprotective molecules. Although preliminary, these results support the rationale and value of future clinical studies with the highest dose of *hf*NPCs in a larger cohort of patients.

## Abstract (web summary)

Phase 1 trial results reveal that intrathecal transplantation of human fetal neural precursor cells in patients with progressive multiple sclerosis is feasible, safe and tolerable.

## Main

Despite the development of several effective treatments that revolutionized the natural history of multiple sclerosis (MS), their advantages are mainly achieved for patients with relapsing-remitting MS (RRMS), while effective therapies for progressive MS (PMS) still represent an unmet need. In PMS, therapeutic mechanisms of action should combine neuroprotection, immunomodulation and regeneration to hamper irreversible disability progression. Most of the ongoing clinical trials for PMS are assessing repurposed drugs with unsatisfactory results due to lack of tailored targets^1^. Neural stem/precursor cells (NPCs) are mitotically active, self-renewing and multipotent cells able to differentiate into astrocytes, oligodendrocytes and neurons and to migrate into specific biological niches or damaged areas promoting functional and structural repair^2^. The paradigm that NPC transplantation acts only by structural cell replacement has been reconsidered. Indeed, NPCs can shape their behavior and fate according to the central nervous system (CNS) microenvironment, maintaining an undifferentiated phenotype^3^. NPCs exert trophic support, immunomodulation and metabolic signaling via paracrine mechanisms and cell-to-cell interaction, the so-called bystander effect, which promotes neuroprotection and tissue repair by re-establishing the functional interactions between neural and glial cells or rousing endogenous neural cells^4–8^. NPC-based therapies may represent a valuable option in PMS, leveraging their extensively pre-clinically assessed multifactorial therapeutic benefits^4,9,10^. In experimental autoimmune encephalomyelitis (EAE), transplanted NPCs showed pathotropic properties migrating to demyelinating areas and inducing a rescue of the functional impairment in transplanted rodents. NPCs promote long-lasting neuroprotection through a bimodal mechanism: differentiating into mature brain cells with a reduction of demyelination, astrogliosis and axonal loss^9^ and exerting trophic support and anti-inflammatory functions maintaining undifferentiated features^4^. NPCs derived from human fetal CNS (*hf*NPCs), due to their replicative capacity in standardized and quality-controlled conditions, can give rise to cell lines that can be exploited in the development of therapeutical strategies of neural transplantation^11^. The therapeutic properties of *hf*NPCs have been confirmed in a non-human primate EAE, which approximates—more closely than rodent models—the characteristics of human MS^10^. Here we report the results of STEMS, the first phase 1 clinical trial evaluating the safety of *hf*NPC transplantation in patients with PMS.

## Trial design

**Fig. 1:**
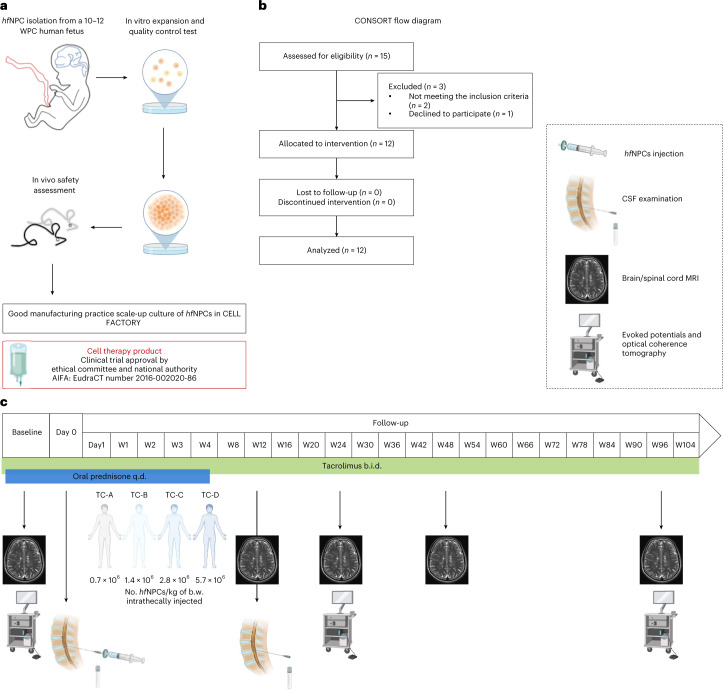
Study design. **a**, The Cell-based Medicinal Product used for transplantation originated from non-immortalized *hf*NPCs (BI-0194-008 cell line) obtained from the telencephalon and diencephalon of a single 10–12 weeks post-conception (WPC) human fetus, after elective pregnancy termination. *hf*NPCs were in vitro expanded and underwent quality control tests. The safety profile of the *hf*NPC cell line was tested in vivo in CD-1 mice immunosuppressed via cyclosporin. Medicinal product manufacturing and release were performed according to GMP conditions. **b**, CONSORT flow diagram. **c**, After enrollment, patients underwent baseline evaluation: neurological examination, blood tests, neurophysiological and neuroradiological assessments. Enrolled patients were divided into four consecutive TCs and received a single intrathecal injection of *hf*NPCs according to the following escalating doses: TC-A: 0.7 × 10^6^ ± 10% cells per kilogram of body weight; TC-B: 1.4 × 10^6^ ± 10% cells per kilogram of body weight; TC-C: 2.8 × 10^6^ ± 10% cells per kilogram of body weight; and TC-D: 5.7 × 10^6^ ± 10% cells per kilogram of body weight. Patients were treated with tacrolimus for 96 weeks after the transplantation (0.05 mg/kg twice daily gradually tapered to a blood level target of 5–10 ng/ml) and oral prednisone 50 mg (starting the day before the procedure and tapered to 0 over 35 days). The safety profile of *hf*NPCs was evaluated via 22 follow-up visits, over 96 weeks after administration to monitor the survival, safety, tolerability and overall changes in the neurological status. A diagnostic lumbar puncture was performed 3 months after transplantation for safety reasons in all treated patients. Some icons of the figure were created with BioRender. b.w., body weight; W, weeks.

STEMS is a prospective, therapeutic exploratory, non-randomized, open-label, single-dose-finding phase 1 clinical trial (NCT03269071, EudraCT2016-002020-86) conducted at San Raffaele Scientific Institute in Milan, Italy (Fig. 1), and approved by the local ethics committee. All participants provided written consent to participate. Because this study was a first-in-human phase 1 trial, we used the standard 3 + 3 design, which is one of the most used designs due to its simplicity of execution and robustness in phase 1 clinical trials^12^. The cell-based medicinal product consisted of *hf*NPCs obtained from a single 10–12 weeks post-conception human fetus (elective abortion) and expanded under Good Manufacturing Practice (GMP) conditions. Patients were consecutively enrolled in four treatment cohorts (TCs), and each of them received a single intrathecal administration of one of the four increasing treatment doses of *hf*NPCs (TC-A: 0.7 × 10^6^, TC-B: 1.4 × 10^6^, TC-C: 2.8 × 10^6^ and TC-D: 5.7 × 10^6^ ± 10% viable cells per kilogram of body weight). Dose scaling was based on the results obtained in preclinical studies on rodent and non-human primates^10^. *hf*NPCs, suspended in 10 ml of injection medium (saline solution containing 0.028% of human serum albumin), were injected in a hospitalized setting through a lumbar puncture, after removal of about 10 ml of cerebrospinal fluid (CSF). The removed CSF was used for chemical–physical, microbiological, oligoclonal band (OB) and research analyses. After the infusion, patients were left in Trendelenburg position for 30 minutes and monitored for 24 hours. To reduce type I hypersensitivity reactions, intravenous pre-medication with 125 mg of methylprednisolone was administered immediately before transplantation. To prevent an unexpected rejection of transplanted *hf*NPCs, despite their relatively low immunogenicity, patients received immune suppression based on tacrolimus^13^ (initial oral dose of 0.05 mg/kg every 12 hours, monitoring serum levels and adjusting dosages to maintain a target of 5–10 ng/ml in peripheral blood) from 7 days before transplantation until week 96 and oral prednisone 50 mg started the day before the procedure and tapered over 35 days. Anti-infective prophylaxis was administered for the duration of the tacrolimus therapy with acyclovir (800 mg twice daily) and cotrimoxazole (800 mg/160 mg, three times per week) associated with folinic rescue (5 mg, three times per week). We treated three patients per TC, monitoring each of them for at least 14 days before proceeding with the treatment of subsequent patient. In the absence of dose-limiting toxicities (DLTs) within each TC, we maintained a 3-month interval before progressing to the higher dose TC. If any patient experienced a DLT in any given TC, the cohort was to be extended to six patients. If more than one DLT occurred, the current dosage was to be considered excessive, and the immediate lower dosage was to be considered the maximum tolerated dose. Key eligibility criteria included a diagnosis of PMS, as per the revised McDonald 2010 criteria, with a progressive course, according to 2013 Lublin phenotypes classification; failure/intolerance to all approved therapies according to the disease course or without any alternative approved therapy; presence of OB in the CSF required for primary PMS (PPMS); evidence of disease progression (increase of ≥0.5 Expanded Disability Status Scale [EDSS] points in the last year); EDSS ≥6.5; age 18–55 years; and disease duration 2–20 years. The primary objective of the study was to evaluate the feasibility, safety and tolerability of intrathecally administered *hf*NPCs in patients with PMS. The *hf*NPCs safety profile was evaluated with 22 follow-up visits over a period of 96 weeks, monitoring for survival, adverse events (AEs) and overall changes in the neurological status. We obtained exploratory hypothesis-generating data about the potential treatment effect of *hf*NPCs (secondary endpoints). Notably, we performed multimodal CSF analyses at baseline and 3 months after transplantation and longitudinally evaluated transplanted patients through clinical, neuroradiological and neurophysiological assessments. The primary safety endpoint was evaluated by descriptive statistics, reporting the number of AEs and the severity within TCs. The longitudinal variations observed in clinical and neurophysiological parameters between month 24 and baseline were tested by an exact Wilcoxon test. The correlations between these changes and the number of injected cells were assessed by a Spearman rank correlation coefficient. For statistically significant correlations, an additional multivariate regression analysis was performed (Methods).

## Results

### Patient characteristics

Between May 2017 and May 2019, 15 patients with PMS were enrolled: 12 patients received the drug product, two left the trial due to immunosuppressive therapy contraindications and one decided to spontaneously leave the trial before transplantation. The last patient last visit was performed in June 2021. Table 1 summarizes the baseline characteristics of the included patients and according to dosage of treatment received.

**Table 1:** Clinical and demographic features

	Treatment cohort (TC)				
	TC-A (0.7 × 10^6^ ± 10% cells/kg)	TC-B (1.4 × 10^6^ ± 10% cells/kg)	TC-C (2.8 × 10^6^ ± 10% cells/kg)	TC-D (5.7 × 10^6^ ± 10% cells/kg)	All TCs
	(*n* = 3)	(*n* = 3)	(*n* = 3)	(*n* = 3)	(*n* = 12)
Female sex, *n* (%)	1 (33.3)	2 (66.7)	3 (100)	2 (66.7)	8 (66.7)
Age (years), mean ± s.d.	50.0 ± 1	50.7 ± 5.9	44.0 ± 4.4	46.3 ± 5.9	47.7 ± 4.9
Disease type					
SPMS, *n* (%)	1 (33.3)	0	2 (66.7)	2 (66.7)	5 (41.7)
PPMS, *n* (%)	2 (66.7)	3 (100)	1 (33.3)	1 (33.3)	7 (58.3)
Baseline EDSS, median (IQR)	8.0 (6.5–8.0)	7.0 (6.5–8.0)	7.5 (7.0–8.0)	6.5 (6.5–7.5)	7.25 (6.5–8.0)
Disease duration (years), mean ± s.d.	18.3 ± 3.1	13 ± 6.6	11.7 ± 1.2	15.3 ± 6.1	14.6 ± 4.9
Previous DMT, *n* (%)					
AZA	1 (33.3)	1 (33.3)	0	0	2 (16.7)
MTX + CTX	1 (33.3)	0	0	0	1 (8.3)
FTY	1 (33.3)	1 (33.3)	1 (33.3)	1 (33.3)	4 (33.3)
RTX/OCR	0	1 (33.3)	1 (33.3)	0	2 (16.7)
CTX	0	0	1 (33.3)	0	1 (8.3)
DMF	0	0	0	2 (66.7)	2 (16.7)
Time from last DMT to transplantation (years), mean ± s.d.					
	3.1 ± 2.4	1.8 ± 1.2	4.2 ± 0.7	2.6 ± 2.3	2.9 ± 1.8
Baseline MRI, mean ± s.d.					
Gd-enhanced T1 lesions (*n*)	0	0	0	0.7 ± 1.2	0.3 ± 0.6
Brain T2 lesion volume (ml)	2.5 ± 0.8	7.4 ± 11.6	6.7 ± 1.4	2.2 ± 0.8	4.7 ± 5.6
Cervical cord T2 lesions (*n*)	4.3 ± 2.5	2.7 ± 1.2	3.7 ± 2.3	2.7 ± 2.1	3.3 ± 1.9
nBV (ml)	1386 ± 102	1421 ± 85	1384 ± 55	1434 ± 64	1406 ± 71
nWMV (ml)	644 ± 85	660 ± 67	646 ± 60	670 ± 38	655 ± 56
nGMV (ml)	742 ± 19	761 ± 35	739 ± 10	764 ± 31	752 ± 25
CSA C1-T1 (mm^2^)	47.3 ± 5.5	61.7 ± 9.8	59.1 ± 3.9	58.9 ± 2.7	56.3 ± 8.4
nLThalV (ml)	9.5 ± 0.7	9.7 ± 1.7	9.3 ± 0.8	9.1 ± 0.7	9.4 ± 0.8
nRThalV (ml)	9.2 ± 0.9	9.5 ± 1.3	9.5 ± 0.7	8.8 ± 0.6	9.2 ± 0.8

SPMS, secondary progressive multiple sclerosis; IQR, interquartile range; DMT, disease-modifying therapy; AZA, azathioprine; MTX, methotrexate; CTX, cyclophosphamide; FTY, fingolimod; RTX, rituximab; OCR, ocrelizumab; DMF, dimethyl fumarate; MRI, magnetic resonance imaging; Gd, gadolinium; nBV, normalized brain volume; nWMV, normalized white matter volume; nGMV, normalized gray matter volume; CSA, cross-sectional area; nLThalV, normalized left thalamic volume; nRThalV, normalized right thalamic volume.

### Primary safety endpoint

Study procedures were well-tolerated, with no notable acute procedural complications. We observed a low-grade, transient headache and neck muscle stiffness, as expected after lumbar puncture. No notable differences in terms of AEs were observed between the TCs (Table 2). No DLTs were reported. Short (within 24 hours) and mid-term (within 14 days) reported AEs were judged as mild and probably or possibly related to the study procedure due to the close time relationship. Nevertheless, they may be also attributable to the disease or to other concomitant drugs and may be unrelated to treatment protocol. CSF examination and magnetic resonance imaging (MRI) 3 months after transplantation ruled out subclinical chronic meningitis, meningeal granulomatosis, obstructive hydrocephalus or intra-axial and extra-axial bleeding. Overall, the observed AEs in the 2-year follow-up were of grade 1 or 2, with the exception of an MS relapse that occurred 78 weeks after the transplantation and led to a clinical worsening (EDSS from 6.5 to 7.5), due to a new enhancing lesion, followed by complete recovery after steroid treatment and physiotherapy. Because the relapse limited, albeit temporarily, the patient’s ambulation, this AE was considered a serious adverse event (SAE), expected (due to patient’s clinical history), and unrelated to the treatment. No grade 3 (or higher) AEs possibly related to the procedure, nor any AEs definitely related to *hf*NPCs, were reported. There were two expected grade 1 AEs characterized by creatinine increase, unlikely related to *hf*NPCs, probably related to the concomitant treatment with tacrolimus and rapidly solved after its permanent discontinuation. Brain and spinal MRI performed 2 months after tacrolimus suspension was negative for signs of host-versus-graft rejection. To date, at least 3 years after the last treated patient, the survival of the patients is 100%.

**Table 2:** Adverse events (AEs) in each TC and in the entire study population (all TCs)

	Treatment cohort (TC)				
	TC-A (0.7 × 10^6^ ± 10% cells/kg)	TC-B (1.4 × 10^6^ ± 10% cells/kg)	TC-C (2.8 × 10^6^ ± 10% cells/kg)	TC-D (5.7 × 10^6^ ± 10% cells/kg)	All TCs
	(*n* = 3)	(*n* = 3)	(*n* = 3)	(*n* = 3)	(*n* = 12)
Number of AEs (%)	14 (31.8)	16 (36.4)	9 (20.4)	5 (11.4)	44
Short-term and mid-term AEs (within 14 days from hfNPC transplantation)					
	6 (60)	1 (10)	3 (30)	0	10
Headache	2^a^	0	1^a^	0	3
Neck pain	1^a^	0	0	0	1
Muscle stiffness	1^a^	0	1^a^	0	2
Hypomagnesemia	1^b^	0	0	0	1
Constipation	1^b^	0	0	0	1
Herpes labialis	0	0	1^b^	0	1
Flu	0	1^b^	0	0	1
Long-term AEs (between 15 days and 96 weeks from hfNPC transplantation)					
	8 (23.4)	15 (44.1)	6 (17.6)	5 (14.7)	34
Gastritis	1^c^	2^c^	0	1^c^	4
Urinary tract infection	1^c^	1^c^	0	1^c^	3
Upper respiratory tract infection	4^c^	3^c^	3^c^	0	10
Hypertransaminasemia	0	2^c^	0	0	2
Skin infection	0	1^c^	0	0	1
Renal colic	0	0	0	1^c^	1
Creatinine increase	1^c^	1^c^	0	0	2
Accidental fall	0	3^d^	0	0	3
Hyperglycemia	0	0	1^d^	0	1
Anemia	0	0	0	1^d^	1
Phlebitis	0	0	2^c^	0	2
MS relapse	0	1^d^ (SAE)	0	0	1
Intestinal polyp	0	1^d^	0	0	1
Renal angiomyolipoma	1^d^	0	0	0	1
Constipation	0	0	0	1^c^	1
Causality of AEs to hfNPC					
Definetely related	0	0	0	0	0
Probably related	4	0	2	0	6
Possibly related	2	1	1	0	4
Unlikely related	7	10	5	4	26
Not related	1	5	1	1	8
Causality of SAE to hfNPCs					
Not related	0	1	0	0	1

^a^ probably related

^b^ possibly related

^c^ unlikely related

^d^ not related

## Secondary exploratory hypothesis-generating endpoints

### Neurologic and neurophysiological outcomes

**Extended Data Fig. 1:**
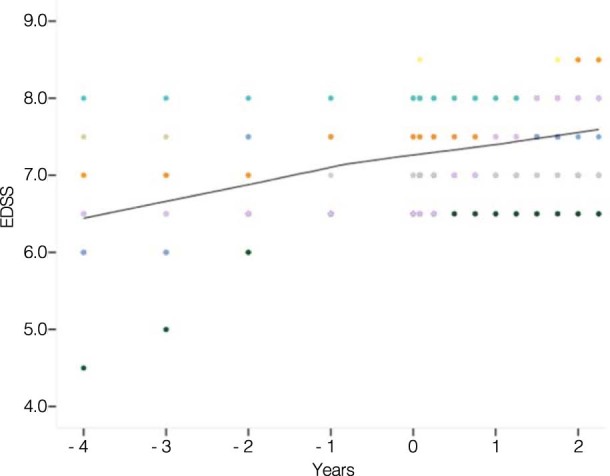
Slope of annual EDSS change before and after transplantation. The slope of mean EDSS rate of change in the 4 years before transplantation (+0.24 EDSS points/year [range 0–0.70]) and in the 2 years after transplant (+0.13 [range 0–0.80]); (p = 0.23, Exact Two-sided Wilcoxon test; p = 0.18 random effect linear model).

The mean rate of EDSS change (slope) in the 4 years before (+0.24 EDSS points per year [range, 0–0.70]) and 2 years after transplantation (+0.13 EDSS points per year [range, 0–0.80]) did not show significant differences (*P* = 0.23, exact Wilcoxon test; *P* = 0.18 random effect linear model; Extended Data Fig. 1). The parameters for motor evoked potentials (MEPs) and sensory evoked potentials (SEPs) in our cohort were already severely impaired at baseline; thus, we adopted a conventional four-point graded ordinal score^14^. Clinical (EDSS, 9-Hole Peg Test (9-HPT) and symbol digit modalities test (SDMT) scores) and neurophysiological (MEP score, SEP score, P100 latency and peripheral retinal nerve fiber layer (pRNFL) thickness) parameters did not show any significant change in the 2-year follow-up. Their longitudinal variations were not significantly associated with the number of injected *hf*NPCs (Table 3).

**Table 3:** Neurological and neurophysiological longitudinal assessments

	Baseline	2-year follow-up	*P* value	Association of 2-year changes vs. number of injected hfNPCs
Clinical assessment				
EDSS score (median [IQR])	7.25 (6.5–8.0)	7.75 (7.0–8.0)	0.06	*r* = 0.28, *P* = 0.37
9-HPT DH, s (mean ± s.d.)	40.1 ± 15.8	97.5 ± 95.6	0.12	*r* = 0.03, *P* > 0.99
9-HPT NDH, s (mean ± s.d.)	46.4 ± 30	71 ± 62.9	0.63	*r* = −0.8, *P* = 0.33
SDMT score (mean ± s.d.)	42.1 ± 110.8	48.6 ± 4.8	0.05	*r* = 0.76, *P* = 0.01^a^
Neurophysiological assessment				
MEP score (median [IQR])	9 (7–10)	8 (7–9)	0.52	*r* = −0.51, *P* = 0.11
SEP score (median [IQR])	8.5 (6–12)	10 (6.5–12)	0.12	*r* = −0.22, *P* = 0.48
P100 Latency, ms (mean ± s.d.)	128.9 ± 14.5	132.2 ± 18.1	0.06	*r* = −0.04, *P* = 0.92
pRNFL thickness, μm (mean ± s.d.)	91.2 ± 11.9	90.7 ± 13.3	0.37	*r* = 0.23, *P* = 0.49

DH, dominant hand; IQR, interquartile range; NDH, non-dominant hand. Two-sided Wilcoxon signed-rank test. Two-sided Spearman correlation coefficient.

^a^ SDMT at the end of follow-up was available for 92% of patients. Two-year variation of SDMT positively correlated with the number of injected cells (*r* = 0.76, *P* = 0.01); however, after adjusting for baseline score, multivariate regression analysis failed to show any statistically significant correlation.

### Plasma biomarkers

No significant changes were detected in tau, neurofilament light (NfL) or ubiquitin C-terminal hydrolase-L1 (UCH-L1) protein levels, whereas plasma levels of glial fibrillary acidic protein (GFAP) increased 2 years after the transplantation (*P* = 0.03) (Supplementary Table 1).

### MRI assessment

**Fig. 2:**
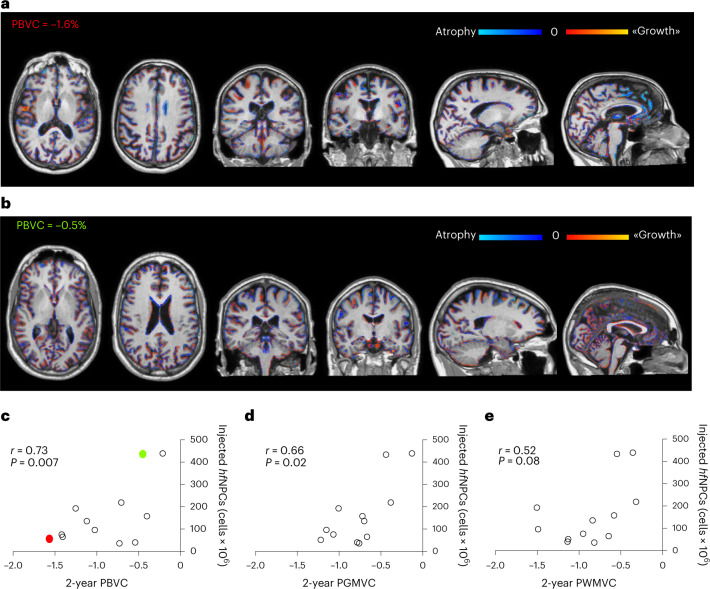
The number of injected *hf*NPCs inversely correlates with brain volume loss. Color-coded areas of brain atrophy (blue) and growth (red) representing PBVC at 2 years from *hf*NPC transplantation in two representative patients, respectively, receiving a low dose (**a**, red in **c**) and high dose (**b**, green in **c**) of *hf*NPCs. **c**, Lower rates of total brain atrophy (percentage of brain volume change [PBVC]) significantly correlate with the number of injected *hf*NPCs (*r* = 0.73, *P* = 0.007). Two-sided Spearman’s correlation test. **d**, Lower rates of GM atrophy (percentage of gray matter volume change [PGMVC]) significantly correlate with the number of injected *hf*NPCs (*r* = 0.66, *P* = 0.02). Two-sided Spearman’s correlation test. **e**, Although not statistically significant, lower rates of brain WM atrophy (percentage of white matter volume change [PWMVC]) seem to correlate with high number of injected *hf*NPCs (*r* = 0.52, *P* = 0.08). Two-sided Spearman’s correlation test.

During the 2-year follow-up, no unexpected findings were detected via MRI. Six patients (50%) developed new brain T2 lesions in the follow-up period (mean increase of T2 lesion volume in the 2-year follow-up ± s.d. = 1.1 ± 0.82 ml), three of which displayed gadolinium-enhancing lesions (GELs). The injected *hf*NPCs number did not correlate with the number (*r* = 0.32, *P* = 0.31) or volume (*r* = 0.24, *P* = 0.45) of new brain T2 lesions nor with the number of GELs (*r* = 0.38, *P* = 0.23). Among patients experiencing MRI activity, patients 004 and 015 had a single punctate new brain T2 lesion without gadolinium enhancement at the end of follow-up (2 years after *hf*NPCs). Patient 013 presented brain GELs from 12 months after transplantation to the end of the follow-up. Patients 008 and 011 had MRI activity before (1 year before and at baseline, respectively) and up to 10 (patient 008) and 24 (patient 011) months after the transplantation. Patient 012 presented MRI activity at baseline without evidence of GELs or new T2 lesions after cell transplantation. Patient 007 experienced a clinical relapse associated with a new dorsal spine GEL 19.5 months after the treatment. Supplementary Table 2 summarizes mean brain tissue and spinal cord atrophy measurements after the 2-year follow-up, reported for the entire cohort and according to the number of injected cells as low-dose (TC-A and TC-B) and high-dose (TC-C and TC-D). A significant difference was found between the annualized rate of gray matter (GM) atrophy for the low-dose versus high-dose groups of patients (*P* = 0.04). Total brain volume changes at 2 years from *hf*NPC transplantation are shown in two representative patients receiving either a low dose (Fig. 2a) versus a high dose (Fig. 2b) of cells. We found that lower rates of total brain atrophy (Fig. 2c) and GM atrophy (Fig. 2d) correlated significantly with the increasing number of injected *hf*NPCs (*r* = 0.73, *P* = 0.007 and r = 0.66, *P* = 0.02), whereas a trend toward a positive correlation was also observed for low rates of brain white matter (WM) atrophy (*r* = 0.52, *P* = 0.08) (Fig. 2e). Multivariate regression analysis confirmed these significant correlations, after adjusting for baseline volumes (whole brain and GM), age, EDSS and 2-year T2 lesion volume change.

### Standard laboratory CSF analysis

Longitudinal CSF chemical–physical analyses showed an increase of proteins and cells 3 months after the transplantation (mean protein ± s.d. 40.8 ± 10.8 mg/dl versus 47.6 ± 13.2 mg/dl, *P* = 0.01; mean cells per microliter ± s.d. 2.6 ± 1.6 versus 7.9 ± 6.9, *P* = 0.01). Link index and IgG CSF levels were unchanged at 3-month follow-up. IgG OBs remained detectable in all the patients after the transplantation. Three months after transplantation, an association was found between CSF protein levels and the number of injected *hf*NPCs (*r* = 0.55, *P* = 0.06), whereas no correlation was found between the number of injected *hf*NPCs and the number of cells found via the chemical-physical analysis after the transplantation (*r* = 0.10, *P* = 0.89).

### CSF donor–host microchimerism

**Extended Data Fig. 2:**
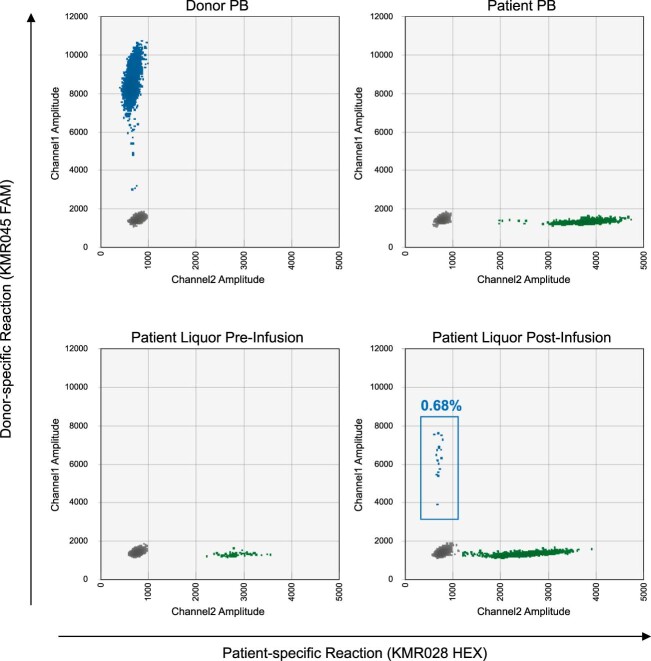
Microchimerism CSF analysis. Patient 012 microchimerism analysis. In the two-dimensional scatter plots, the y-axis indicates detection of genomes bearing the donor-specific polymorphism (KMR045 marker, blue dots), and the x-axis measures the detection of genomes bearing the patient-specific polymorphism (KMR028 marker, blue dots). The upper panels display results from the germline control samples (DNA from donor and patient’s peripheral blood samples), and the lower panels display results from CSF samples collected before treatment and 3 months after transplantation.

In five patients (001, 007, 011, 012 and 013), it was possible to extract from CSF samples collected 3 months after treatment an adequate amount of genomic DNA to perform microchimerism analysis (median, 5.9 ng; range, 0.1–7.2). Upon optimization of a droplet digital PCR (ddPCR)-based method to ensure high sensitivity and accuracy, in one of these patients (012) the presence of donor-derived cells, amounting to 0.68% of the analyzed material, was detected (Extended Data Fig. 2).

### Inflammatory, proteomic and metabolomic CSF profiling

**Fig. 3:**
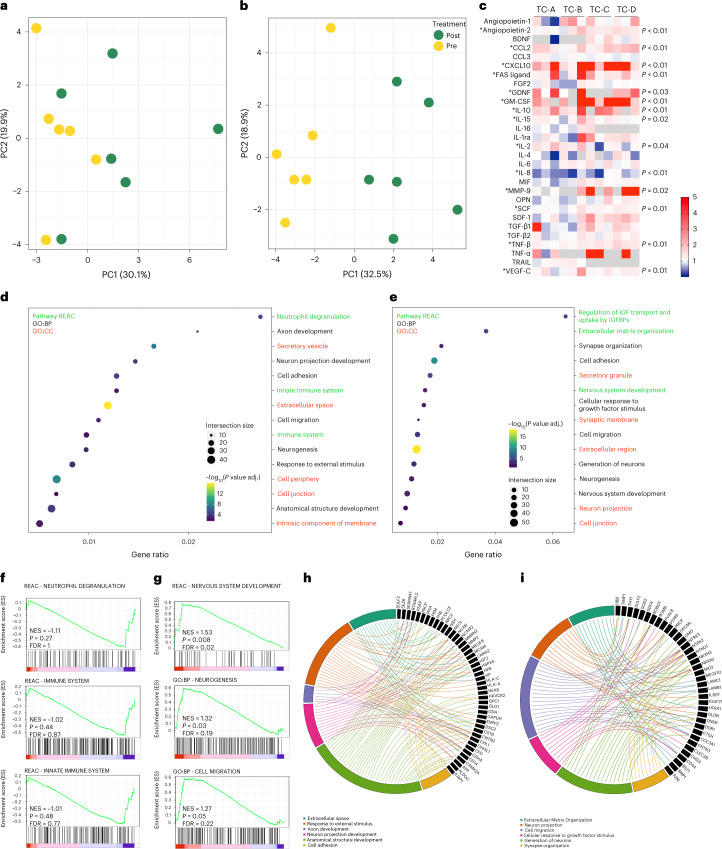
CSF immunological and proteomic profile. PCA of the detectable CSF analytes evaluated at baseline (yellow) and 3 months after (green) transplantation in the low-dose (n.6) (**a**) and high-dose (n.6) (**b**) groups. **a**, In the low-dose group, the first two principal components, PC1 and PC2, explained 30.1% and 19.9% of the variation, respectively. **b**, In the high-dose group, PC1 and PC2, explained 32.5% and 18.9% of the variation, respectively, with a clear distinction between baseline and 3-month follow-up samples. **c**, Heat map representing the fold change (red, upregulation; blue, downregulation) of CSF analyte (*y* axis) between baseline and 3-month follow-up for each patient (*x* axis). Differences in the expression of each analyte were calculated by a non-parametric exact two-sided Wilcoxon test. * indicates statistically significant difference (exact *P* < 0.05). **d**,**e**, Pathway *e*nrichment analysis on CSF differentially expressed proteins after transplantation in the ‘low-dose’ (**d**) and ‘high-dose’ groups (**e**). Dot plots of enriched Reactome (REAC) pathways and Gene Ontology (GO) terms (REAC in green labels, GO:BP in black labels and GO:CC in red labels) of biological interest selected among the top enriched terms (FDR < 0.05). The *x* axis represents the ‘rich factor’ (gene ratio) of each term. The dots’ size and color represent the gene number (intersection size) and the adjusted *P* value (−log_10_[*P* value adj.]), respectively. **f**,**g**, Curves of pre-ranked GSEA for selected datasets, showing the profile of the running enrichment score (ES) and positions of gene set members on the rank-ordered list. The GSEA curves of neutrophil degranulation, immune system and innate immune system pathways show a trend for a negative enrichment in the low-dose group (**f**). In the high-dose group, the GSEA curves of nervous system development pathway, and neurogenesis and cell migration GO terms show significant positive enrichment (**g**). Comparisons were performed using a two-sided paired Studentʼs *t*-test. Significance levels were adjusted with the Benjamini–Hochberg multi-test correction. **h**,**i**, Chord plots of enriched pathways of biological interest, selected among the top 20 enriched, in the low-dose (**h**) and high-dose (**i**) groups.

**Extended Data Fig. 3:**
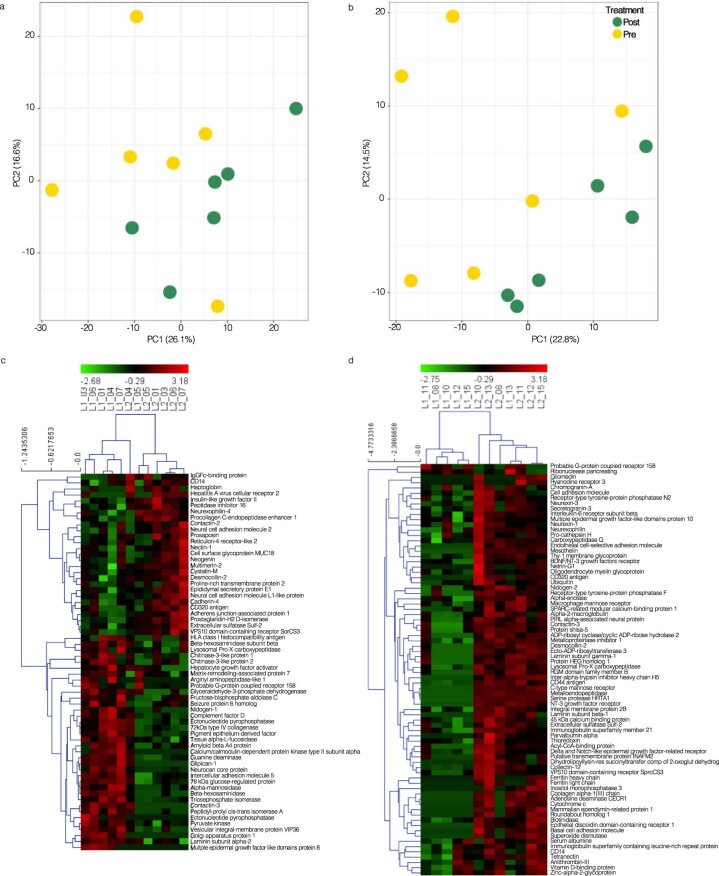
CSF proteomic profile at baseline and 3 months after transplantation. Principal component analysis (PCA) on the CSF proteins evaluated at baseline and three months after transplantation in the low-dose (n. 6) (**a**) and high-dose (n. 6) (**b**) groups. **a**) In the low-dose group, the first two principal components (PC1 and PC2) explained 26.1 and 16.6% of the variation, respectively, with a modest distinction of the two time points. **b**) In the high-dose group, the first two principal components (PC1 and PC2) explained the 22.8 and 14.5% of the variation, respectively, highlighting a distinction of the baseline from the 3-month follow-up samples. **c**, **d**) Unsupervised hierarchical clustering of CSF differentially expressed proteins between baseline and 3-month follow-up and the related heatmap in the low-dose (**c**) and high-dose (**d**) groups of patients.

**Extended Data Fig. 4:**
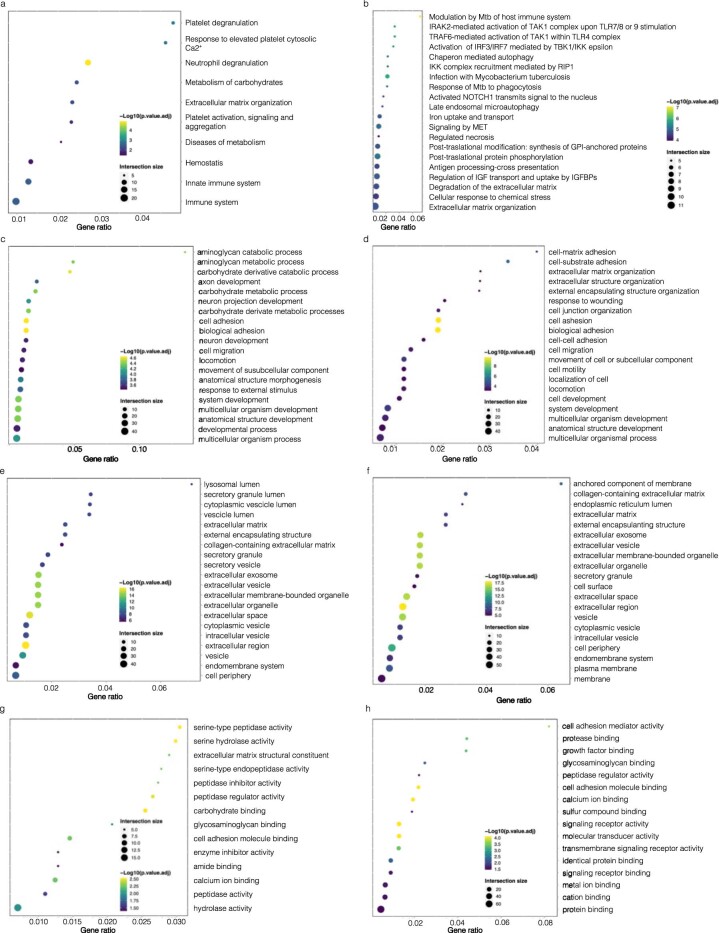
Top enriched pathways and gene ontology terms in CSF differentially expressed proteins. The top enriched (FDR < 0.05) Reactome (REAC) pathways (**a**, **b**), gene ontology (GO) terms concerning biological process (GO:BP) (**c**, **d**), to molecular function (GO:MF) (**e**, **f**), and to cellular component (GO:CC) (**g**, **h**). The low-dose group (n. 6) is represented on the left, the high-dose group (n. 6) on the right. On the y-axes the enriched pathways are listed, while the x-axis represents the ‘rich factor’ (gene ratio) of each term. The size and colour of dots represent the gene number (intersection size) and the adjusted p-value (reported as -Log10[p.value.adj]), respectively. Comparisons were performed using a two-sided paired Student T-test. Significance levels were adjusted with the Benjamini-Hochberg multitest correction.

**Extended Data Fig. 5:**
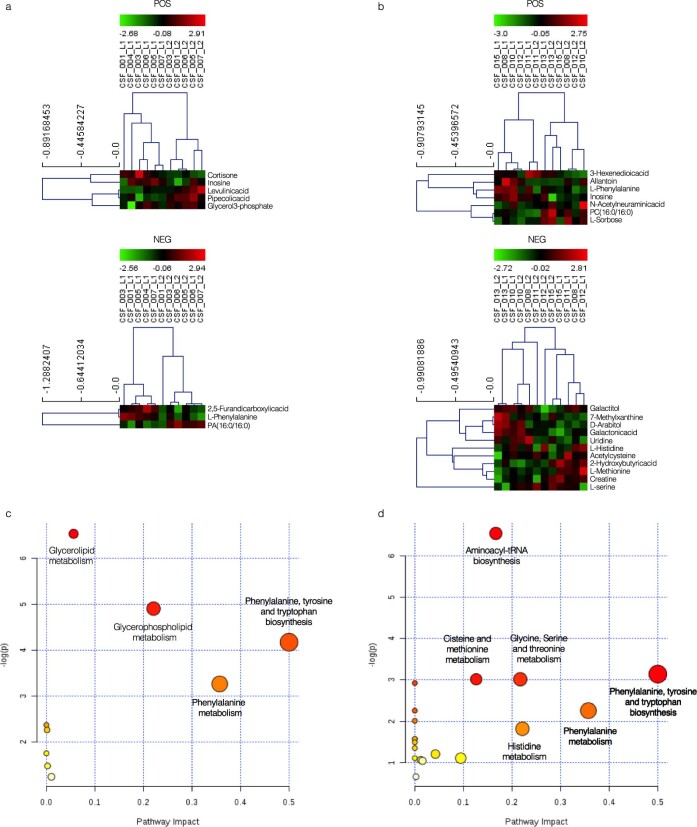
CSF metabolomic profile at baseline and 3 months after transplantation. **a**, **b**) Unsupervised hierarchical clustering of CSF differentially expressed metabolites in positive (POS) and negative (NEG) mass spectrometry modalities between baseline and 3-month follow-up and the related heatmap in the low-dose (n. 6) (**a**) and high-dose (n. 6) (**b**) groups of patients. The pathway analysis performed on these metabolites revealed the involvement of pathways related to phenylalanine, tyrosine, and tryptophan biosynthesis and metabolism in the low-dose (**c**) and high-dose (**d**) groups of patients. Comparisons were performed using a two-sided paired Student T-test. Significance levels were adjusted with the Benjamini-Hochberg multitest correction.

Of 46 tested cytokines, chemokines and growth factors, 17 remained below the detectable range at baseline and after the transplantation (CCL4, CCL11, EGF, G-CSF, IFN-y, IL-1b, IL-5, IL-7, IL-12p40, IL-12p70, IL-17, IL-22, LIF, PDGF-AB, PDGF-BB, RANTES and TGF-B3). We performed a principal component analysis (PCA) on all the detectable analytes evaluated at baseline and 3 months after transplantation; the first two principal components (PC1 and PC2) represent the main axes of variation within these data and explained 30.1% and 19.9% of variation, respectively, for the low-dose group (Fig. 3a) and the 32.5% and 18.9% for the high-dose group (Fig. 3b). The scatter plots of these components highlight the algorithm’s ability to distinguish the baseline from the 3-month follow-up samples, showing a more evident CSF immunological profile change in the high-dose group of patients (Fig. 3b). After transplantation, we identified significant changes in the expression level of angiopoietin-2 (mean level ± s.d. at baseline 114.3 ± 36 pg/ml versus 3-month follow-up 157.5 ± 53.7 pg/ml, *P* = 0.002 Wilcoxon test), CCL-2 (296.2 ± 107.7 pg/ml versus 490.1 ± 170.4 pg/ml, *P* = 0.001), CXCL10 (119.1 ± 62.2 pg/ml versus 665.6 ± 628.2 pg/ml, *P* < 0.001), FAS ligand (6 ± 2.3 pg/ml versus 11.1 ± 5.9 pg/ml, *P* = 0.003), GDNF (0.2 ± 0.1 pg/ml versus 0.4 ± 0.2 pg/ml, *P* = 0.03), GM-CSF (4.8 ± 2.9 pg/ml versus 27 ± 18.5 pg/ml, *P* = 0.002), IL-10 (2.2 ± 1 pg/ml versus 5.7 ± 3.2 pg/ml, *P* = 0.002), IL-15 (4.1 ± 1.9 pg/ml versus 5 ± 2.2 pg/ml, *P* = 0.02), IL-2 (59.8 ± 58.9 pg/ml versus 96.7 ± 103.8 pg/ml, *P* = 0.04), IL-8 (130.4 ± 107.8 pg/ml versus 53 ± 16.1 pg/ml, *P* = 0.002), MMP-9 (95.9 ± 116.6 pg/ml versus 238.7 ± 116.7 pg/ml, *P* = 0.02), SCF (23.2 ± 4.7 pg/ml versus 27.3 ± 7.4 pg/ml, *P* = 0.01), TNF-β (1.7 ± 7.1 pg/ml versus 9 ± 8.2 pg/ml, *P* = 0.01) and VEGF-C (137.8 ± 53.2 pg/ml versus 175.5 ± 67.5 pg/ml, *P* = 0.01) (Fig. 3c). Next, we performed a systematic identification and quantification of the CSF proteins through proteomic analysis. A PCA performed on all the detectable proteins showed the CSF profile change after transplantation (Extended Data Fig. 3a,b). Of 707 and 714 identified and quantified proteins in the low-dose and high-dose groups, 63 and 78, respectively, resulted in being significantly differentially expressed between paired CSF samples at baseline and 3 months after transplant (*P* < 0.05, paired Student’s *t*-test), as reported in the relative heat maps in Extended Data Fig. 3c,d. To establish the biological relevance of these changes, we submitted to gProfiler2 R library the list of differentially expressed proteins and measured their enrichment with pathways in Reactome collection and Gene Ontology (GO) terms. The top enriched (false discovery rate (FDR) < 0.05) pathways and GO terms with respect to biological process (GO:BP), molecular function (GO:MF) and cellular component (GO:CC) are represented in Extended Data Fig. 4. Enriched pathways and terms of biological interest were selected among the top enriched list for the low-dose and high-dose groups, respectively (Fig. 3d,e). In the low-dose group, the pathway’s analysis showed an enrichment of immune system (gene ratio = 0.01, FDR = 0.003), innate immune system (gene ratio = 0.01, FDR = 0.003) and neutrophils degranulation (gene ratio = 0.01, FDR < 0.001) pathways (not enriched in the high-dose group). Notably, GO analyses showed the involvement of biological domain like extracellular matrix organization (in low-dose gene ratio = 0.02, FDR = 0.005; in high-dose gene ratio = 0.03, FDR = 0.005), cell migration (in low-dose and high-dose gene ratio = 0.01, FDR < 0.001) and adhesion (in low-dose gene ratio = 0.01, FDR < 0.001; in high-dose gene ratio = 0.02, FDR < 0.001), axon development (in low-dose gene ratio = 0.02, FDR < 0.001; in high-dose gene ratio = 0.02, FDR = 0.01), neuron projection morphogenesis (in high-dose gene ratio = 0.01, FDR = 0.03), cellular response to growth factors (in high-dose gene ratio = 0.02, FDR = 0.01) and synapse organization (in high-dose gene ratio = 0.02, FDR = 0.002). To investigate the CSF proteomic changes in more detail, we performed a pre-ranked gene set enrichment analysis (GSEA) that demonstrated a general downregulation trend for pathways related to innate immunity (neutrophil degranulation: NES (normalized enrichment score) = −1.11, *P* = 0.27, FDR = 1; immune system: NES = −1.02, *P* = 0.44, FDR = 0.87; and innate immune system: NES = −1.01, *P* = 0.48, FDR = 0.77) (Fig. 3f). Interestingly the GSEA analysis showed an overall upregulation for protein sets involved in nervous system development (NES = 1.53, *P* = 0.008, FDR = 0.02), neurogenesis (NES = 1.32, *P* = 0.03, FDR = 0.19) and cell migration (NES = 1.27, *P* = 0.05, FDR = 0.22) (Fig. 3g). The poor statistical power of the GSEA analysis could be related to the limited number of analyzed samples per group. The significantly enriched pathways identified as of biological interest have proven to be closely interconnected (Fig. 3h,i). Before and after *hf*NPC transplantation, untargeted metabolomics analysis of CSF samples identified only 8 (of 128) and 18 (of 100) metabolites as significantly differentially represented (*P* < 0.05, paired Student’s *t*-test), for the low-dose and high-dose groups, respectively (Extended Data Fig. 5a,b). The pathway analysis performed on these metabolites, in both the low-dose and high-dose groups, revealed involvement of metabolites related to aromatic amino acids metabolites (phenylalanine, tyrosine and tryptophan) (Extended Data Fig. 5c,d), a profile that could reflect the patient disease phenotype rather than a treatment effect, as previously suggested^15^.

## Discussion

To our knowledge, STEMS is the first phase 1 clinical trial assessing the safety and maximum tolerated dose of intrathecally transplanted *hf*NPCs in patients with PMS. The primary outcome - safety - was reached with no severe adverse reactions related to the cell product at 2-year follow-up. Although quite preliminary, given the study’s uncontrolled design and small sample size, mechanistic studies revealed some interesting results that could support the speculation that *hf*NPCs might play a neuroprotective role, acting as a source of trophic factors and immunological modulators. We found increased levels of CSF proteins, able to foster a neuroprotective remodeling of the CNS microenvironment, in addition to a significantly reduced rate of the whole brain and GM volume loss, which correlated with the number of injected *hf*NPCs.

In terms of safety, neither AEs of grade 3 or higher possibly related to the procedure, nor AEs definitely related to *hf*NPC treatment, were reported. All the observed AEs were mild (grade 1) or moderate (grade 2), except one case of MS relapse that was defined as a SAE, given the worsening (though transitory) of the EDSS score. This relapse occurred 78 weeks after the transplantation in a patient previously treated with fingolimod, which was started in 2012, after experiencing an inflammatory reactivation, and was interrupted 6 months before the enrollment in our study. Therefore, the SAE was considered not related to the experimental cell therapy approach. In addition to this patient experiencing a combined clinical and radiological relapse, five additional patients presented new MRI lesions during the 2-year follow-up. These were not considered AEs because they were not associated with clinical relapses and were evaluated as events related to the disease course. Overall, the rate of patients with new T2 lesions observed during the follow-up (50%) was unexpectedly high in patients with severe longstanding progressive disease. To explore a possible correlation between MRI activity and our treatment, we performed a correlation analysis that does not show a clear relationship between the number of injected *hf*NPCs and the number and volume of new T2 lesions. Although we did not find any correlation, given the lower statistical power, we cannot rule out the possibility of increased MRI lesion activity resulting from transplantation, and this observation needs to be deeply analyzed in future clinical studies. One patient who received the highest dosage of *hf*NPCs, and for whom the transplanted cells were found in the CSF 3 months after transplantation by microchimerism analysis, showed GEL at baseline, but no new lesions were observed during the 2-year follow-up. Although STEMS was designed to evaluate the safety, tolerability and feasibility of *hf*NPC transplantation in patients with PMS, we have also collected and evaluated, as exploratory hypothesis-generating analyses, preliminary data about disability, cognition, atrophy and CSF parameters. In our cohort of patients, we observed a trend toward clinical worsening in terms of EDSS and 9-HPT scores. Despite not being statistically significant, the slope of the mean annual EDSS change seems to show a trend for lower variation after the transplantation compared to the 4 years before. However, this result should be analyzed cautiously. The EDSS scores in the years preceding the study enrollment were collected retrospectively. Moreover, our patients could present an inclusion bias because it was required to meet the inclusion criterion of clinical worsening in the previous 12 months. This inclusion criterion might have made it more difficult to detect further clinical deterioration via the EDSS, and we cannot exclude the regression to the mean phenomenon. Furthermore, it is well established that the EDSS is a non-linear ordinal scale, and, in cases of high disability, it has low sensitivity to patients’ changes^16^. In addition, the 9-HPT score, which is more sensitive than EDSS in identifying a worsening in more advanced stages of the disease, showed a worsening in the 2-year follow-up.

Despite pRNFL thickness being considered a potential biomarker of neuroaxonal loss, representing a reliable objective tool to assess novel neuroprotective therapies in PMS^17^, our patients did not show significant pRNFL variation over time. Cognitive dysfunction with impaired information processing speed significantly contributes to disability in PMS^18^. Our patients showed a significant improvement in SDMT scores, one of the tests used to evaluate cognitive processing speed^19^. Patients receiving higher doses of *hf*NPCs were also those with worse baseline SDMT, and multivariate analysis adjusted for baseline SDMT did not show a correlation between SDMT score variation and the number of injected *hf*NPCs. We cannot exclude that the improvement we found is related to the regression to mean phenomenon^20^. Furthermore, practice effect could represent a bias in the repeated cognitive assessment.

MRI brain and GM volume measurements represent neurodegeneration biomarkers in patients with MS^21^ and outcome measurements in several phase 2 clinical trials^22,23^. Degeneration and consequent GM atrophy are more pronounced in advanced phases of the disease, being closely associated with worsening of disability in all forms of MS^24,25^. Interestingly, in our exploratory analysis, we found a statistically significant correlation between the percentage of annual brain and GM volume change, evaluated in the 2-year follow-up, and the number of injected *hf*NPCs. Brain volume changes in MS could be the result of different mechanisms, including not only tissue loss or regeneration but also changes in non-tissue components, such as fluid shift resulting from inflammation within active lesions. The focal edema subsequent to new lesion formation may mask volume loss, especially in the WM where inflammation is more pronounced^26^. However, the correlation between brain atrophy and injected *hf*NPCs remained robust in multivariate regression analysis, after adjusting for baseline volumes, age, EDSS and T2 lesion volume change, making the above-mentioned mechanisms unlikely in our cohort. Moreover, brain atrophy is a chronic process, and 2-year changes (a typical follow-up in a PMS clinical trial) may not suffice to properly assess this slowly evolving phenomenon. Because volumetric MRI measurements depend on several factors, which make the interpretation of brain atrophy measurements challenging^27^, the use of accurate and robust image analysis tools and visual inspection of the results represent a strength of our study. Although these results need to be confirmed on a larger cohort of patients, the multivariate analysis confirms the strength of the correlation restraining the possibility of the influence of confounding factors.

CSF immunological and proteomic analyses were performed at baseline and 3 months after transplantation. We found an upregulation of some trophic factors (that is, GDNF, VEGF-C and SCF) and immune-related molecules (that is, FAS ligand and IL-10) and the downregulation of some pro-inflammatory chemokines, such as IL-8. Although we cannot exclude that our results might have been biased by the concomitant immunomodulatory treatments, it is possible to hypothesize that transplanted *hf*NPCs might have played a neuroprotective role. GDNF is a neurotrophic factor enhancing the survival, neurite outgrowth and differentiation of distinct populations of neurons^28^. VEGF-C converts quiescent neural stem cells into progenitor cells, stimulating hippocampal neurogenesis and neuronal plasticity^29^. FAS ligand is critically involved in the NPC-mediated pro-apoptotic effect on encephalitogenic Th1 and Th17 lymphocytes, as previously demonstrated in EAE mice^4,30^.

We also found an upregulation of cytokines and chemokines, such as IL-15 and GM-CSF. Although pro-inflammatory in nature, these molecules have been also shown to exert pro-regenerative functions. IL-15 regulates NPC proliferation/differentiation, and its deficiency leads to an impaired generation of neuroblasts in the subventricular zone (SVZ)–rostral migratory stream axis^31^. Similarly, GM-CSF has neurotrophic and neurogenic activity, inhibiting both NPC and neuronal apoptosis^32^. However, we cannot exclude that GM-CSF increase might be due to blood-borne CNS-infiltrating monocytes/macrophages driving an inflammatory response that, in turn, might also explain the MRI activity observed in 50% of our patients who received transplantation.

Moreover, our proteomic analysis supports a possible pro-regenerative role of *hf*NPCs. Compared to the baseline, the CSF proteomic pathway analysis highlighted an enrichment of the extracellular matrix (ECM) organization pathway and the membrane-ECM interaction pathway after the treatment; such evidence might reflect possible cross-talk between *hf*NPCs and the blood-brain barrier (BBB) in treated patients, as previously demonstrated in preclinical studies^9,10^. Finally, the GO performed on CSF differentially expressed proteins showed a positive enrichment of cellular pathways responding to growth factors as well as a clear involvement of neuroplasticity-associated pathways, such as membrane adhesion, axon development and cell projection morphogenesis. Our results are in line with previous evidence in murine models of Alzheimer’s disease showing that NPC transplantation reduced memory deficits through the promotion of neurogenesis, synaptic plasticity and dendritic stability^33,34^. Although encouraging and consistent with preclinical data, a confounding effect in interpreting the CSF analyses might be attributed to concomitant treatments and to the study’s uncontrolled design.

Our previously published preclinical results clearly demonstrated that cells might retain viability and keep exerting their bystander effect even months after transplantation. However, we cannot speculate on how long the cells remain viable and potentially efficacious because the study design (with few timepoints and no long-term follow up) did not allow for CSF samples to be obtained beyond 3 months after transplantation. Moreover, the positive result of the microchimeric assay in one patient - one of three who received the highest dose of cells - though of interest, has only a descriptive value and could represent a cue for subsequent studies.

In conclusion, our study showed that *hf*NPC therapy in PMS is feasible, safe and tolerable. Preliminary exploratory results on brain volume changes and CSF remodeling warrant future clinical studies with the highest dose of cells in a larger cohort of patients. It remains to be established whether *hf*NPC transplantation might have the potential to exert a long-lasting bystander effect in humans, impacting the clinical course of the progressive phase of MS.

## Methods

### Ethical regulations

The study was conducted in compliance with the Declaration of Helsinki and Good Clinical Practice. It was approved by the San Raffaele Scientific Institute (Milan, Italy) ethics committee and was authorized by the AIFA (Italian Medicines Agency). All participants provided written consent to participate. None of the study participants received compensation for participation in the study. The trial is registered at ClinicalTrials.gov (NCT03269071) and the European Union Clinical Trials Register (EudraCT no. 2016-002020-86).

### *hf*NPC manufacturing

The cell-based medicinal product (CBMP) used for the transplant originated from non-immortalized *hf*NPCs, BI-0194-008 cell line, obtained from the telencephalon and diencephalon of a single 10/12-week post-conception human fetus, after elective pregnancy termination. Serological tests for HIV (type I and II), HTLV I and II, hepatitis B and C and *Treponema pallidum* performed on maternal blood samples resulted negative. Human tissue was provided by ‘Banca Italiana - Fondazione IRCCS CA’ GRANDA Ospedale Maggiore Policlinico di Milano’. Permission to use human fetal CNS tissue was granted by San Raffaele Hospital’s Ethics Committee. Tissue procurement was in accordance with the Declaration of Helsinki and in agreement with the ethical guidelines of the European Network for Transplantation (NECTAR). Fresh human fetal brain tissue was mechanically chopped and enzymatically dissociated with Trypsin LONZA (BE17-161E) 1:5 in growth medium for 5–10 minutes at 37 °C, 5% O_2_ and 5% CO_2_. After washing with 10% Australian FBS (10099133, Gibco) in fresh medium and centrifuging for 15 minutes at 200*g*, the cells were plated in T25 flasks in NeuroCult-XF Proliferation Medium (05761, STEMCELL Technologies) with rh-EGF (236-GMP-01M, R&D Systems) and rh-bFGF (233-GMP-01M, R&D Systems), both at 20 ng/ml, at 37 °C, 5% O_2_ and 5% CO_2_. After 15–25 days, neurospheres or subconfluent adherent cells were enzymatically dissociated with StemPro Accutase Cell Dissociation Reagent (A1110501, Gibco) and replated at clonal density (20–25,000 cells per cm^2^) in NeuroCult-XF Proliferation Medium with rh-EGF and rh-bFGF after the evaluation of cell count and viability. The last step was repeated every 10–15 days. BI-0194-008 *hf*NPCs were extensively characterized for the expression of stem cell markers (Nestin, CD133 and SOX2) and pathotropic markers, such as cell surface adhesion molecules (CD44), chemokine receptors (CXCR4) and integrins (CD29 and integrin β1 chain) by flow cytometry (FC) in proliferating cells (≥10% positive cells). For the purpose of batch release we have chosen the expression of Nestin, CD133 and SOX2 markers as the proof of evidence of the undifferentiated state of our *hf*NPCs lines^35–37^ (Supplementary Fig. 1). Cells were fixed for 10 minutes in 2% paraformaldehyde and then permeabilized and blocked with 5% fetal serum in PBS, 0.1% Triton on ice for 15 minutes; anti-CD133 (130-098-829, Miltenyi Biotec, 1:30) and anti-Nestin (IC1259F, R&D Systems, 1:10), anti-CD44 (21330443, Immunotools, 1:10), anti-CXCR4 (555974, BD Bioscience, 1:10) and anti-CD29 (130-123-935, Miltenyi Biotec, 1:10) were added and incubated for 30 minutes on ice. For SOX2 staining, cells were fixed and permeabilized using the FoxP3 Staining Buffer Kit (130-093-142, Miltenyi Biotec). Cells were then incubated with the anti-SOX2 antibody (130-104-994, Miltenyi Biotec, 1:10) or with the corresponding REA Control (130-113-438, Miltenyi Biotec). After staining, cells were washed with PBS and acquired on a BD FACSCalibur (BD Biosciences) and analyzed by BD CellQuest Pro or FACSDiva (BD Biosciences). To check for the multipotentiality of *hf*NPCs, their differentiation potential into glial or neuronal progenies upon growth factor (GF) removal was investigated in vitro. GFAP, Tuj1 and O4 were quantified by immunofluorescence (IF) on differentiated cells. In total, 70,000 cells were seeded in the center of a 13-mm-diameter coverslip covered with Corning Matrigel Growth Factor Reduced (GFR) (354230). Cells were kept in differentiating medium for 7–10 days and, after differentiation, were fixed for 10 minutes in 4% paraformaldehyde (P6148, Sigma-Aldrich), washed and incubated for 1 hour, at room temperature, in a blocking solution containing 10% donkey serum (D9663, Sigma-Aldrich). For GFAP and Tuj1 staining, the blocking also contained Triton 0.1% (X-100, Sigma-Aldrich). Primary antibodies (anti-GFAP, 1:500, Z0334, Dako; anti-Tuji1, 1:1,000, 802001, BioLegend; and anti-O4, 1:200, O7139, Sigma-Aldrich) were added and left for overnight incubation at 4 °C. After washing, the appropriate Alexa Fluor-labeled secondary antibody (Life Technologies) was added for 1 hour (Alexa Fluor 488, 1:1,000, A-21206, for GFAP and Tuj1 and Alexa Fluor 488, 1:1,000, A-21042, for O4). Nuclei were stained with 4′,6-diamidino-2-phenylindole (5 mg/ml in dimethylformamide) 1:25,000 in PBS (D1306, Life Technologies), and slides were mounted using fluorescent mounting medium (S3023, Agilent Technologies). Finally, the slides were visualized under a confocal microscope (Leica TCS SP5 Laser Scanning Confocal).

### Safety profile of the *hf*NPC cell line (BI-0194-008)

Toxicity induced by *hf*NPCs after a single intracerebroventricular (ICV) administration was evaluated in vivo in CD-1 mice. CD-1 mice were treated with cyclosporin (10 mg/kg/day from day 2 to the end of the study) to minimize immune response. The study was performed at ‘Accelera S.r.l – Nerviano’ in compliance with Italian Legislative Decree (D.L. no. 50 dated 2 March 2007, as published in G.U. no. 86 of 13 April 2007) and with organization for Economic Co-operation and Development (OECD) Principles of Good Laboratory Practice (GLP) (C(97) 186 (Final)). Animals were stratified based on body weights and distributed into the three experimental groups using random number tables (Fisher and Yates): (1) untreated control, (2) ICV vehicle (PBS) administration + daily oral cyclosporin and (3) ICV *hf*NPCs administration (1 × 10^6^ cells per mouse) + daily oral cyclosporin (Supplementary Table 3). The animals underwent clinical evaluation daily; body weight was recorded weekly; and food intake was measured at 2-week intervals. Hematology and serum chemistry (blood count, urea, creatinine, aspartate amino transferase, alanine amino transferase, alkaline phosphatase, total protein, albumin, globulin, glucose, triglycerides and albumin/globulin ratio) were performed on days 30, 60 and 92. At the end of the experimental phase (day 92), the animals were sacrificed, and a full necropsy was performed. Organ weights and gross observations data were collected from all experimental groups. Histological examination of selected organs and tissues from animals from the experimental group receiving oral cyclosporin and *hf*NPCs or the vehicle was performed at the end of the observation period (days 92–95). The animals’ environmental conditions were continuously monitored, and deviations from set parameters were recorded. All the environmental conditions, as well as all the procedures adopted throughout the study for housing and handling the animals, were in strict compliance with EC and Italian Guidelines for Laboratory Animal Welfare. No treatment-related mortality occurred during the study, nor were any toxicologically relevant changes reflected by the clinical signs, body weights, food intake or clinical or pathological examinations. No treatment-related findings were noted at gross or histological examinations in mice treated with *hf*NPCs. In conclusion, *hf*NPCs given as single ICV administration in CD-1 mice followed by a 90-day observation period did not elicit any adverse events or signs of toxicity.

### *hf*NPC expansion

Cells used for transplant in patients derived from the expansion of the BI-0194-008 cell line performed at ‘Laboratorio di Terapia Cellulare e Genica Stefano Verri - ASST - Monza, Ospedale San Gerardo’ under GMP conditions with standard operating procedures (Supplementary Fig. 2). The starting material to generate the working cell bank (WCB) was a batch of the master cell bank (MCB) that consisted of a six-passage single expansion of *hf*NPC BI-0194-008 cell line. After the BI-0194-008 cell expansion, a total of approximately 1.9 × 10^9^ cells were recovered and resuspended in ice-cold freezing medium (complete medium, added with 20 ng/ml of *rh*-EGF and *rh*-bFGF + 10% DMSO). The cell suspension was frozen (46 × 10^6^ cells per vial) according to the standard operating procedure (POS-310304). The MCB was then tested for identity and safety (Charles River Laboratories) (Supplementary Table 4). Next, we obtained the WCB by a sequential expansion of the MCB. Cells were plated at a density of 30 × 10^3^ ± 5 × 10^3^ cells per cm^2^, and neurospheres were dissociated when they reached approximately 100–150 μm in diameter. After six steps, cells were harvested, washed and stored as described for the MCB (Supplementary Table 4). The Drug Substance Process validation was assessed and evaluated on the basis of a standard operating procedure (POS-200815-179) and production batch record. For this purpose, four large-scale GMP-compliant expansion runs were performed at the ‘Laboratorio Verri’. The first run was performed to obtain the MCB (as previously described), whereas the subsequent three runs (generated from distinct aliquots of the MCB as starting material) were performed to assess and validate most of quality control assays and to assess the stability plan. Aliquots of the cell suspension were collected at different timepoints, and viability was immediately determined by trypan blue dye exclusion test, to identify the maximum time delay from thawing to injection. The threshold of viability (45%) is maintained up to 5 hours after thawing; thus, 4 hours were identified as the maximum time delay from thawing to injection. When a new patient, enrolled in the clinical protocol, was ready for treatment, cryopreserved WCB vials were thawed. The freezing medium was discarded, and cells were re-suspended in 10 ml of injection medium (10 ml of saline solution containing 0.028% of human serum albumin). Before administration, a viability assay and sterility test were performed (Supplementary Table 4). Release criteria for clinical use included: the mean viability ≥45% (assessed by trypan blue vital dye exclusion), undifferentiated *hf*NPCs state (CD133 ≥10%, SOX2 ≥10% and Nestin ≥10%), sterility of the sample, absence of mycoplasma and bacterial endotoxin (gel-clot endotoxins assay) and lack of any genomic copy number changes as assessed with 1-Mb-resolution bacterial artificial chromosome array comparative genomic hybridization. Additionally, an identity test for human nature and specific DNA identity (barcode sequencing and DNA fingerprint) was performed. Any additional chromosomal alterations that might have occurred during the in vitro expansion were ruled out by cytogenetic analyses (Karyotype), performed according to standard methods in the ‘Laboratorio di Emato-Oncologia Pediatrica - Fondazione Tettamanti – Ospedale San Gerardo – Monza’. The threshold of 45% cell viability seems low considering that standard values for cell therapy products usually are >70–80%. However, our final drug product originates directly from a thawed WCB, which, in turn, was expanded from a thawed MCB; thus, the freezing and thawing process clearly may affect cell viability. During the validation process of the final drug product, we obtained a mean cell viability after thawing of 55.15% (range, 45.23– 66.50%). Based on these results, we chose the lowest value of 45% as stringent and reliable threshold for batch release. The dose of final drug product to be administered to the patients was calculated as number of viable cells per kilogram of body weight.

### Inclusion and exclusion criteria

Recruited patients were required to meet the following inclusion criteria: signature of the Informed Consent Form by the patient or patients’ legal guardians; age 18–55 years; diagnosis of PMS as per the revised McDonald 2010 criteria with a progressive course according to 2013 Lublin phenotypes classification with failure or intolerance to all approved therapies according to the disease course or without any alternative approved therapy; disease duration 2–20 years; EDSS ≥6.5; evidence of progression of the disease defined by an increase of ≥0.5 EDSS points in the last 12 months; and presence of OBs in the CSF required for PPMS. Exclusion criteria included: any active or chronic infection or disease other than MS including, but not limited to, HIV1-2, hepatitis B, hepatitis C, tuberculosis or immune deficiency syndromes; treatment with any immunosuppressive therapy within the 3 months before screening; treatment with interferon-beta, glatiramer acetate or corticosteroids within the 30 days before screening; a relapse occurred during the 30 days before screening; contraindications for or intolerance to any medications, treatments and procedures used in the study; pregnant or breastfeeding women or women of childbearing age who are not willing to use a contraceptive method effective for the entire duration of the study; and any condition that, in the opinion of the investigator, would preclude study participation.

### Primary endpoint

To address the primary objective, we considered short-term (0–24 hours), mid-term (day 1 to day 14) and long-term (day 15 to week 96) safety endpoints. The short-term and mid-term safety endpoints aimed to exclude risks related to the procedure of cell injection, such as aseptic meningitis or obstructive hydrocephalus, and allergic reactions to the medicinal product. Long-term safety endpoints had the primarily purpose of excluding the presence of infections due the possible non-sterility of the cell product (a risk minimized by the extensive in vitro examinations), tumor formation (risk minimized by the in vivo evidence obtained by infusion of supra maximal doses of cells in immunosuppressed mice) as well as changes in neurological status that would not be expected based on the disease course. The documented AE severity was classified according the Common Terminology Criteria for Adverse Events (CTCAE) (grade 1=mild; grade 2=moderate; grade 3=severe; grade 4=life-threatening; grade 5=death), and the attribution to the investigational medicinal product (unrelated, unlikely, possibly, probably, definitely) was defined accordingly to National Cancer Institute guidelines of AE reporting requirements. SAE was defined as any untoward expected medical occurrence or effect that, at any dose, results in death, that is life-threatening, that requires hospitalization or prolongation of existing hospitalization, that results in persistent or significant disability or incapacity or that is a congenital anomaly or birth defect. The safety profile of *hf*NPCs was evaluated with 22 follow-up visits, over a period of 96 weeks after administration, monitoring for survival, safety, tolerability and overall changes in the neurological status. Patients were evaluated by a complete physical examination, clinical laboratory tests and instrumental follow-up (electrocardiogram and chest X-ray at 1 year and 2 years and abdominal ultrasound at 2 years). A diagnostic lumbar puncture was performed for safety reasons 3 months after the *hf*NPC injection in all the treated patients to rule out the presence of infective or aseptic meningitis or obstructive hydrocephalus.

### Exploratory endpoints

In addition to safety endpoints, we evaluated exploratory endpoints to obtain preliminary data on the possible *hf*NPC effect in treated patients with PMS. In particular, we evaluated:

I. Disability and cognitive progression assessed through:
  - Expanded Disability Status Scale (EDSS)
  - 9-Hole Peg Test (9-HPT) in dominant hand (DH) and non-dominant hand (NDH)
  - Symbol Digit Modalities Test (SDMT)
II. Longitudinal changes in:
  - Motor-evoked potentials (MEP)
  - Somatosensory-evoked potentials (SEP)
  - Visual-evoked potentials (VEP)
  - Optical coherence tomography (OCT): peripheral retinal nerve fiber layer (pRNFL) thickness
III. Brain and spinal cord MRI measures:
  - Number and volume of brain and spinal cord T2 lesions, number of new lesions and changes in T2 lesion volume at weeks 48 and 96
  - Total brain and spinal cord gadolinium-enhancing lesions at baseline, weeks 48 and week 96
  - Brain and spinal cord annualized atrophy rates, as defined by percentage in brain volume and spinal cord cross-sectional area changes from baseline to week 96
IV. Longitudinal plasma measurement of NfL, GFAP, UCH-L1 and TAU
V. CSF analysis at baseline and 3 months after the transplantation:
  - Donor-recipient chimerism
  - Immunological profile
  - Metabolomic and proteomic profile

### EDSS, 9-HPT and SDMT

The EDSS is based on a standard neurological examination, incorporating seven functional systems (visual, pyramidal, cerebellar, brainstem, sensory, bowel and bladder and cerebral) rated and stored as functional system scores (FSSs). Each FSS is an ordinal clinical rating scale ranging from 0 to 5 or 6. These ratings are then used in combination with ambulation capacity to determine the EDSS score that ranges from 0 (normal) to 10 (death due to MS). Sustained disability progression is defined as an increase of >0.5 EDSS points in the last 12 months. This change must not be attributable to another etiology (that is, fever, concurrent illness, MS relapse or concomitant medication), and it must be confirmed at a regularly scheduled visit after at least 12 weeks after the initial disease progression. The neurological examination with EDSS assessment was performed at baseline, at day −1 and at 1, 3, 6, 9, 12, 15, 18, 21 and 24 months. The 9-HPT evaluates upper extremities and coordination. To conduct this timed test, the patients are seated with a peg board in front of them. The board has nine holes on one side and a shallow round dish on the other side, and the patients are asked to place the pegs into each hole on the peg board, one at a time, and remove them again one at a time, while the test speed is measured. The patients were tested twice for each arm, the dominant arm first. An average time was calculated separately for the dominant and non-dominant hands. The SDMT quantifies the speed of information processing, by asking the patient to associate symbols and numbers as quickly as possible. The patient’s score is estimated as the number of correct associations produced in 90 seconds. 9-HPT and SDMT were evaluated at baseline and at 6, 12, 18 and 24 months.

### MEP and OCT

MEP, SEP, VEP and OCT were performed at baseline and at 6, 12 and 24 months. MEPs of the four limbs were obtained using a Cadwell MS10 magnetic stimulator with a round coil (outer diameter, 12 cm). The coil was placed tangentially to the scalp, with its center over the vertex. Patients were asked to slightly contract the target muscles (abductor pollicis brevis and abductor hallucis) at about 20% of the maximum voluntary effort to facilitate motor responses. Spinal roots were stimulated at C6–C7 and L4– L5 spaces while recording from the same muscles. Central motor conduction time was measured as the difference between total and peripheral motor conduction time. SEPs were obtained on electrical stimulation of the median nerve at the wrist for the upper limb and at the tibial nerve for the lower limb. Latencies of the main peripheral, spinal and cortical components were measured, and central conduction time was calculated as the difference between cortical and spinal latencies (for tibial nerve SEP, latencies were corrected by height). VEPs to reversal achromatic checks (each subtending 60, 30 and 15 minutes of visual angle (min arc)) were recorded over Oz of the 10–20 international EEG system, with Cz as the reference. Latency and amplitude of the P100 component were measured. For all evoked potential modalities, latencies and amplitudes were measured. OCT was performed using spectral domain Spectralis OCT (Heidelberg Engineering, software version 5.8). Standard pRNFL was measured on a standard 3.5-Ø-mm circle scan protocol centered on the optic disc by the scanning technician. Global pRNFL thickness was automatically measured through the pre-installed software provided by the manufacturer. pRNFL thinning and p100 latency increase may also occur as a result of MS-associated optic neuritis (MS-ON). Patients were categorized into those with prior history of MS-ON and those without MS-ON (non-MS-ON) and assessed accordingly. For patients with a history of MS-ON in one eye, the eye without prior MS-ON was studied. For patients with a history of MS-ON in both eyes or no optic neuritis (ON) at all, the pRNFL and P100 mean of both eyes was considered. MEP and SEP abnormalities were quantified according to a conventional four-point graded ordinal score (0 = normal; 1 = increased latency; 2 = increased latency plus morphological abnormalities of a major component; and 3 = absence of a major component). For each modality, the evoked potential score was the sum of the scores in the two sides and in upper and lower arms (from 0 to 12), with higher values representing a more severe evoked potential involvement^14^.

### Brain and spinal cord MRI acquisition

Using a 1.5-Tesla MR scanner system (Achieva dStream, Philips Medical Systems), the brain and spinal cord MRI scans were acquired at baseline (before transplantation) and at 3, 6, 12, and 24 months after the transplantation:

I. Brain sequences:
  - 3D fluid-attenuated inversion recovery (FLAIR) (repetition time (TR) = 4,800 ms, echo time (TE) = 277 ms, inversion time (TI) = 1,660 ms, echo train length (ETL) = 171, flip angle (FA) = 90°, field of view (FOV) = 230 × 230 × 184 mm^3^, 160 sagittal slices, voxel size = 1.15 × 1.15 × 1.15 mm^3^, matrix = 200 × 200 × 160, number of signal averages (NSA) = 2);
  - 2D T2-weighted turbo spin echo (TSE) (TR = 4,446 ms, TE = 100 ms, ETL = 15, FA = 90°, FOV = 230 × 192 mm^2^, 22 axial slices; slice thickness= 5 mm, gap= 1 mm, pixel size = 0.6 × 0.9 mm^2^, matrix= 384 × 224, NSA = 3);
  - 2D T2-weighted fast field echo (FFE) (TR = 698 ms, TE = 23 ms, FA = 13°, FOV = 256 × 256 mm^2^, 22 axial slices, slice thickness = 5 mm; gap= 1 mm, pixel size = 1.0 × 1.25 mm^2^, matrix = 256 × 204, NSA = 2);
  - Diffusion-weighted imaging (DWI) (TR = 4,043 ms, TE = 65 ms, FA = 90°, FOV = 240 × 240 mm^2^, 22 axial slices, slice thickness = 4 mm; gap = 0.4 mm, n° b-factors: 2 (max b-factor: 1,000), 3 DW directions, pixel size = 2.05 × 2.56 mm^2^; matrix = 116 × 94, NSA = 1);
  - 3D T1-weighted turbo field echo (TFE) (TR = 8 ms, TE = 3.7 ms, TI = 1,000 ms, ETL = 171, FA = 8°, FOV = 256 × 256 × 192 mm^3^, 160 sagittal slices, voxel size = 1.0 × 1.0 × 1.2 mm^3^, matrix = 256 × 256 × 160, NSA = 1);
  - Post-contrast 2D T1-weighted TSE (TR = 580 ms, TE = 12 ms, FA = 69°, FOV = 230 × 183 mm^2^, 22 axial slices, slice thickness = 5 mm, gap = 1 mm, pixel size= 0.85 × 1.12 mm^2^; matrix = 272 × 164, NSA = 2), 5 minutes after the injection of gadobutrol 0.1 ml/kg;
  - Post-contrast 3D T1-weighted TFE, with the same acquisition parameters as for sequence (e), 5 minutes after the injection of gadobutrol 0.1 ml/kg.
II. Spinal cord sequences:
  - Cervical and dorsal 2D short-tau inversion recovery (STIR) (TR = 2,500 ms, TE = 50 ms, TI = 170 ms, ETL = 15, FA = 90°, FOV = 380 × 380 mm^2^, 15 sagittal slices, slice thickness = 3 mm, gap = 0.3 mm, pixel size= 0.9 × 1.25 mm^2^, matrix = 424 × 296, NSA = 2);
  - Cervical cord 3D T1-weighted TFE scan (TR = 8.2 ms, TE = 3.8 ms, TI = 1,100 ms, ETL = 255, FA = 8°, matrix = 256 × 255, FOV = 250 × 250 × 65 mm^3^, 65 sagittal slices, voxel size = 0.98 × 0.98 × 1.0 mm^3^, matrix = 256 × 256 × 65, NSA = 1);
  - Cervical and dorsal 2D T1-weighted TSE (TR = 400 ms, TE = 7.4 ms, ETL = 4, FA = 90°, FOV = 380 × 380 mm^2^, 15 sagittal slices, slice thickness = 3 mm, gap = 0.3 mm, pixel size = 1.0 × 1.25 mm^2^, matrix = 380 × 304, NSA = 2);
  - Crvical and dorsal 2D T2-weighted TSE (TR = 3,748 ms, TE = 120 ms, ETL = 50, FA = 90°, FOV = 380 × 380 mm^2^, 15 sagittal slices, slice thickness =3 mm, gap = 0.3 mm, pixel size = 0.9 × 1.25 mm^2^, matrix = 424 × 300, NSA = 3);
  - Post-contrast cervical and dorsal 2D T1-weighted TSE, with the same acquisition parameters as for sequence (c), 5 min after injection of gadobutrol 0.1 ml kg^−1^, after brain 2D T1-weighted TSE and 3D T1-weighted post-gadolinium sequences.

For all brain scans, the slices were positioned to run parallel to a line that joins the most infero-anterior and infero-posterior parts of the corpus callosum (AC–PC line), covering the entire brain from the skull base to the vertex, with careful repositioning during the follow-up scan.

### Lesion segmentation, count and volume assessment

Focal brain T2-hyperintense WM lesions were identified by a fully automated deep learning approach using co-registered 3D FLAIR and 3D T1-weighted images as inputs (nicMSlesions, relase 0.2, Python version 2.7)^38^. Lesion segmentation was reviewed by an experienced observer, blinded to patients’ identity, and, for a few cases, manual editing was used to remove false-positive lesions. Segmentation of brain gadolinium-enhancing lesions, spinal cord T2-hyperintense lesions and spinal cord gadolinium-enhancing lesions was manually performed on brain pre-contrast and post-contrast 3D T1-weighted, cervical and dorsal spinal cord STIR and spinal cord post-contrast 2D T1-weighted sequences, respectively. On follow-up scans, the number of new brain and spinal cord T2-hyperintense and gadolinium-enhancing lesions were counted as well as brain and spinal cord lesions showing a >50% volume enlargement compared to the previous timepoint.

### Whole and regional brain tissue volume analyses

At baseline, whole brain, GM and WM volumes were measured on 3D T1-weighted images after lesion filling^39^ using the SIENAX version 2.6, FSL 5.0.1 software. The percentage brain volume changes (PBVCs) were estimated at each follow-up timepoint versus baseline visit using FSL SIENA software^40^. The percentage of GM and WM volume changes were calculated using the segmentation tool and pairwise longitudinal registration in the Statistical Parametric Mapping (SPM) toolbox version 12, MATLAB version 8.0, to segment the tissues and quantify volume changes from images of the Jacobian determinants derived from the non-linear registration between two visits^41^. The thalami were automatically segmented from the 3D T1-weighted images using FSL 5.0.1 FIRST software^42^. The result of the segmentation was visually checked; then, the volume was extracted and normalized for head size using the scaling factor previously assessed within FSL SIENAx software.

### Cervical cord area analysis

Baseline and follow-up cervical cord area measurements were performed on 3D T1-weighted scans with the active surface segmentation method available in Jim software package (Version 7, Xinapse Systems)^43^. The semi-automatic method was run to estimate the cervical cord outline from the top of the odontoid process of the axis to the inferior border of C7. After visualizing the active surface output (and editing the output when needed), cross-sectional cord area (CSA) was calculated by dividing the cervical cord volume by the cervical cord length. Annualized CSA changes were calculated between timepoints.

### Plasma research analysis

Plasma samples collected immediately before and 1, 3, 6, 12 and 24 months after transplant from the 12 treated patients were analyzed for NF-L, GFAP, Tau and UCHL-1 content using Simoa Human Neurology 4-Plex A (N4PA, 102153) digital immunoassay on an HD-1 single-molecule array (Simoa) instrument according to instructions from the manufacturer (Quanterix). The measurements were performed by a technician blinded to clinical data, in one round of experiment using one batch of reagents and with baseline and follow-up samples analyzed side-by-side. Plasma samples were diluted four times. This is a two-step immunoassay, in which target antibody coated paramagnetic beads are combined with sample and biotinylated detector antibody in the same incubation. Target molecules present in the sample are captured by the antibody-coated beads and bind with the biotinylated antibody detector simultaneously. After a wash, a conjugate of streptavidin-ß-galactosidase (SBG) is mixed with the beads. SBG binds to the biotinylated detector antibodies, resulting in enzyme labeling of the captured target. After a final wash, the beads are resuspended in a resorufin ß-d-galactopyranoside (RGP) substrate solution and transferred to the Simoa Disc. Individual beads are then sealed within microwells in the array. If the target has been captured and labeled on the bead, ß-galactosidase hydrolyzes the RGP substrate in the microwell into a fluorescent product that provides the signal for measurement. A single-labeled target molecule results in sufficient fluorescent signal in 30 seconds to be detected and counted by the Simoa optical system.

### Genomic DNA extraction and microchimerism analysis by ddPCR

Genomic DNA was extracted from peripheral blood (PB) and cerebrospinal fluid (CSF) samples using the Qiamp DNA Blood or Qiamp DNA micro kits (51104 and 56304, Qiagen), checked for purity using a NanoDrop spectrophotometer (Thermo Fisher Scientific), quantified using the Qubit dsDNA HS Assay Kit (Q32851) according to the manufacturer’s protocol and then stored at −20 °C for further analyses. To identify informative polymorphisms for the subsequent microchimerism analysis, donor-derived and patient-derived genomic DNA were screened using the quantitative PCR (qPCR)-based KMRtype genotyping assay (8841781, GenDx), according to the manufacturer’s recommendations. The kit covers 39 bi-allelic markers distributed over 20 different chromosomes, multiplexed in ten different assays. Based on the results, donor-specific (positive in the donor and negative in the patient) and patient-specific (positive in the patient and negative in the donor) markers were selected (Supplementary Fig. 3a,b). Donor microchimerism analysis was performed employing customized fluorescent KMRtrack assays (8842982, kindly provided by GenDx) and adapting the protocol to the QX100 Droplet Digital PCR system (Bio-Rad). In brief, selected FAM and HEX KMRtrack assays (20×), each specific for donor or recipient genomic markers, were used in combination for each sample, together with the 2× ddPCR Supermix for Probes (Bio-Rad) and up to 10 ng of genomic DNA, in a total volume of 20 μl. Immediately after droplet generation by the Automated Droplet Generator (Bio-Rad), the amplification reaction was performed using a T100 thermal cycler from Bio-Rad with the following PCR cycling protocol: 95 °C 10 minutes; 95 °C for 10 minutes, followed by 40 cycles of amplification (94 °C for 30 minutes and 62 °C for 1 minute), ending with 98 °C for 10 minutes. Donor-specific and patient-specific signals in each droplet were detected by the QX200 Droplet Reader (Bio-Rad). Lastly, ddPCR data were analyzed using QuantaSoft software version 1.7 (Bio-Rad), pooling results from replicates and deriving donor chimerism according the formula: (donor-specific signal / (donor-specific signal + patient-specific signal)) × 100. Chimerism was corrected for the zygosity status of the donor and patient genomic markers targeted by the selected KMRtype assays, inferred by comparing them with the signal from a non-polymorphic genetic marker located in the TTC5 gene (dHsaCP2506310 and dHsaCP2506733; Bio-Rad). To validate custom assays, patient-specific artificial chimeric curves were generated, diluting donor DNA into patients to reproduce the 50%, 10%, 1%, 0.1% and 0.01% donor chimerism situations. Next, 10 ng of each DNA mixture were tested by ddPCR using the appropriate combination of donor-specific and patient-specific reactions, selected in accordance with the screening results (Supplementary Fig. 3a,b). A high correspondence between expected and detected donor chimerism was observed (Supplementary Fig. 3c), with the ability in four of five chimeric curves tested to detect donor chimerism down to the 0.1% dilution. For the relevant PB and CSF samples, a median of 7.2 ng (range, 4.3–10.5 ng) and 5.9 ng (range, 0.1–7.2 ng) of genomic DNA was tested.

### Luminex analysis

CSF samples, obtained at baseline and 3 months after transplantation, were analyzed in two different experiments, dividing samples of patients who received a low dose (TC-A and TC-B) and patients who received a high dose (TC-C and TC-D) of *hf*NPCs. Reagents for Luminex assays were developed by R&D Systems (LXSAHM-05, LXSAHM-34, LXSAHM-04, LTGM100, LTGM200 and LTGM300). The following biomarkers were analyzed according to reports in the literature: ANGIPOIETIN-1, ANGIPOIETIN-2, BDNF, EGF, EOTAXIN, FAS LIGAND, FGFbasic, G-CSF, GDNF, GM-CSF, IFNy, IL-10, IL-12p40, IL-12p70, IL-15, IL-16, IL-17A, IL-1B, IL-1ra, IL-2, IL-22, IL-4, IL-5, IL-6, IL-7, IL-8, IP-10, LIF, LT-alpha, MCP-1, MIF, MIP-1α, MIP-1B, MMP-9, OSTEOPONTIN, PDGF-AB, PDGF-BB, RANTES, SCF, SDF-1α, TGFβ1, TGFβ2, TGFβ3, TNFα, TRAIL and VEGF-C. Unfiltered CSF was diluted using diluents supplied by the manufacturer. Each 96-well plate included seven-fold serial dilutions of standards tested in duplicate. Assays were performed according to the manufacturer’s magnetic Luminex screening assay protocol. In brief, a microparticle cocktail, diluted CSF and biomarker standards were added to a 96-well plate. After a 2-hour incubation, plates were washed, and a biotin antibody cocktail was added. After a 1-hour incubation, plates were washed, and streptavidin–phycoerythrin (PE) was added for 30 minutes, followed by a final wash and resuspension in wash buffer. All incubations were done at room temperature on a microplate shaker. Plates were read immediately on the Luminex 100 instrument, and raw data were analyzed using bio-plex manager version 6.0 software. Analytes were defined detectable when their value was above the detection limit in more than 50% of samples.

### Metabolomic and proteomic analysis

CSF samples, obtained at baseline and 3 months after transplantation, were analyzed in two different experiments, dividing samples of patients who received a low dose (TC-A and TC-B) and patients who received a high dose (TC-C and TC-D) of *hf*NPCs. CSF samples were spun at 300*g* to separate cells and immediately snap-frozen on dry ice and stored at −80 °C. Samples were tested either at protein or metabolite level.

### Label-free quantitative proteomics analysis

To better characterize the relevant proteins, 250 µl of CSF was depleted using a Seppro IgY 14 spin column (SEP010, Sigma-Aldrich), thus allowing the removal of the 14 highly abundant proteins (Albumin, α1-Antitrypsin, IgG, IgA, IgM, Transferrin, Haptoglobin, β2-Macroglobulin, Fibrinogen, Complement C3, α 1-Acid Glycoprotein [Orosomucoid], HDL [Apolipoproteins A-I and A-II] and LDL [mainly Apolipoprotein B]), according to the manufacturer’s instructions. The recovered supernatant was analyzed to determine total protein concentration using the BCA protein assay kit from Pierce (23227, Thermo Fisher Scientific) and BSA as standard. Then, 20 µg of total proteins from each sample was in-solution digested (1,4-dithioerythritol, D8255, Sigma-Aldrich; iodoacetamide, I1149, Sigma-Aldrich; ammonium bicarbonate, O9830, Sigma-Aldrich; trypsin sequencing grade, 11418475001, Roche-Merck). Aliquots of the samples containing tryptic peptides were desalted using StageTip C18 (87784, Thermo Fisher Scientific) and analyzed by nano-scale liquid chromatographic tandem mass spectrometry (nLC–MS/MS) using a Q-Exactive mass spectrometer (Thermo Fisher Scientific) equipped with a nano-electrospray ion source (Proxeon Biosystems) and an nUPLC Easy nLC 1000 (Proxeon Biosystems). Peptide separations occurred on a homemade (75 µm i.d., 15-cm-long) reverse phase silica capillary column (PF360-75-10-N-5, New Objective), packed with 1.9-µm ReproSil-Pur 120 C18-AQ (r119.aq, Dr. Maisch GmbH). A gradient of eluents A (distilled water with 0.1% v/v formic acid, 85171, Thermo Fisher Scientific) and B (acetonitrile with 0.1% v/v formic acid, 85175, Thermo Fisher Scientific) was used to achieve separation (300 nl/min flow rate), from 2% B to 40% B in 88 minutes. Full scan spectra were acquired with the lock-mass option, resolution set to 70,000 and mass range from *m*/*z* 300 to *m*/z 2,000. The ten most intense doubly and triply charged ions were selected and fragmented. All MS/MS samples were analyzed using Mascot (version 2.6, Matrix Science) search engine to search the human_proteome 20180912 (95,106 sequences and 37,690,780 residues) for the low-dose dataset and the human_proteome 20191113 (96,456 sequences and 38,349,854 residues) for the high-dose dataset. Searches were performed with the following parameters: trypsin as proteolytic enzyme; two missed cleavages allowed; carbamidomethylation on cysteine as fixed modification; protein N-terminus acetylation, methionine oxidation as variable modifications; and mass tolerance was set to 5 p.p.m. and to 0.02 Da for precursor and fragment ions, respectively. To quantify proteins, the raw data were loaded into MaxQuant^44^ software version 1.6.1.0. Label-free protein quantification was based on the intensities of precursors. The experiments were performed in technical triplicates.

### Untargeted metabolomics analysis

Metabolites were extracted from 100 µl of CSF using 1.5 ml of 50% methanol (414832, Carlo Erba)/30% acetonitrile (412342, Carlo Erba)/20% water (412112, Carlo Erba) extraction buffer^45^. The extraction was carried out at 4 °C for 15 minutes with shaking. The samples were then centrifuged at 16,000*g* at 4 °C for 10 minutes. The surnatants were transferred to mass spectrometry vials and directly analyzed using the UPLC 1290 (Agilent Technologies) coupled to the TripleTOF 5600+ mass spectrometer (SCIEX). MS acquisition was done in both positive and negative mode. Chromatographic separations occurred on a Sequant pZIC-HILIC (150 × 2.1 mm, 150442, Merck Millipore) capillary column, packed with 5-µm polymer. A gradient of eluents A (acetonitrile, 412342, Carlo Erba) and B ((NH_4_)_2_CO_3_ 20 mM (1.59504, Merck Millipore) + 0.1% NH_4_OH (221228, Sigma-Aldrich)) was used to achieve separation (200 µl/min flow rate), from 20% B to 80% B in 15 minutes; the column temperature was set at 45 °C, and the autosampler was set at 4 °C. Full scan spectra were acquired in the mass range from *m*/*z* 75 to *m*/*z* 1,000. The eight most intense ions were selected and fragmented. The source parameters were: Gas 1: 33 psi, Gas 2: 58 psi, Curtain gas: 25 psi, Temperature: 500 °C, ISVF (IonSpray Voltage Floating): 5,500 V (positive) and 4,500 V (negative), DP: 80 V and CE: 35 V with a spread of 10 V. Bioinformatics tools (MasterView version 1.1, MeV version 4.9.0 and MetaboAnalyst version 4.0) were applied for statistical validation and metabolomic data interpretation to identify the significant CSF metabolites and the altered metabolic pathways in the experimental groups. Accurate Mass Metabolite Spectral Library version2.0 (SCIEX) was used for metabolite identification.

### Statistical analysis and data representation

The EDSS course over time was compared within patients by estimating the yearly rate of EDSS change before and after transplantation. The patients’ specific slopes were compared by a non-parametric exact Wilcoxon test and running a random effect model adjusting for the within-patient correlation. Changes of all the MRI and clinical parameters were estimated over 2 years and summarized as mean or median changes (with standard deviations and ranges), according to the distributions of the data. The within-patient changes between year 2 and baseline visits were compared for all the neurophysiological, MRI and laboratory parameters by a non-parametric exact Wilcoxon test. All the correlations were assessed using Spearman’s correlation coefficient. The relationship of brain atrophy data with the number of cells infused was evaluated by a multivariable ANOVA model, after checking for normality assumptions. Owing to the exploratory nature of the analyses research endpoints, no corrections for multiple testing were performed, and *P* values must be considered only as descriptive.

For proteomic and metabolomic analysis, the entire dataset of identified and quantified analytes was subjected to two-sided paired Student’s *t*-test. Significance levels were adjusted with the Benjamini–Hochberg multi-test correction. A hierarchical clustering analysis was performed on the significantly differently expressed proteins, using MeV software version 4_9_0 (ref. 46). Enrichment analysis was performed with the Gprofiler2 R library (version 0.2.1, R version 3.6.3), starting from the list of genes associated with the differentially expressed proteins and testing their overlap with pathways annotated in the Reaction collection and from the three gene ontologies (cellular components GO:CC, biological processes GO:BP and molecular functions GO:MF). The gene ratio was of the number of differentially expressed proteins interrogated as genes annotated in a pathway to the number of all the genes annotated in the pathway. The most interesting pathways among the ones identified as significant were further inspected using the Preranked GSEA algorithm, version 4.1.0 (ref. 47): even if this method is not suited for proteomics data, it can be a powerful tool to inspect the overall direction of the observed deregulation and whether it is mainly driven by upregulated or downregulated genes. Luminex results were reported via heat map using the GENE-E platform (GENE-E). Hierarchical clustering was displayed as a heat map with the dendrogram using Morpheus analysis software (https://software.broadinstitute.org/morpheus/), with Spearman rank correlation. The PCA was carried out using the prcomp R package: data were preliminarily centered and scaled to get zero-mean and unit variance on each protein. Data were analyzed with the SPSS statistical package (version 24.0) and with GraphPad Prism (version 8.0, GraphPad Software). Heat map representation was performed in R. *P* values less than 0.05 were considered statistically significant

### Reporting summary

Further information on research design is available in the Nature Portfolio Reporting Summary linked to this article.

## Online content

Any methods, additional references, Nature Portfolio reporting summaries, source data, extended data, supplementary information, acknowledgements, peer review information; details of author contributions and competing interests; and statements of data and code availability are available at 10.1038/s41591-022-02097-3.

## Supplementary information

Supplementary Information Supplementary Figs. 1–3 and Supplementary Tables 1–4

Reporting Summary

## Data Availability

Pseudonymized participant data, including baseline characteristics and results of primary and exploratory endpoint analyses reported in this article, can be shared in compliance with current data protection regulations by the European Union. Data sharing requires a current and positive vote by the requestor’s competent ethics committee. All proposals should be directed to the corresponding author. The mass spectrometry proteomics data have been deposited to the ProteomeXchange Consortium via the PRIDE partner repository with the datasets identifier (1) PXD034840 and 10.6019/PXD034840 for the low-dose experiment and (2) PXD034846 and 10.6019/PXD034846 for the high-dose experiment.
