# Supplementary material for: Neural stem cell transplantation in patients with progressive multiple sclerosis: an open-label, phase 1 study
Source: Nat Med. 2023 Jan 9;29(1):75–85. doi: 10.1038/s41591-022-02097-3 (PMC9873560; doi:10.1038/s41591-022-02097-3)
Supplement: Supplementary file 1 — Supplementary Figs. 1–3 and Supplementary Tables 1–4 [file 41591_2022_2097_MOESM1_ESM.pdf]

nature medicine

Article

https://doi.org/10.1038/s41591-022-02097-3

Neural stem cell transplantation in patients with progressive multiple sclerosis: an open-label, phase 1 study

In the format provided by the authors and unedited

Supplementary information

Supplementary Figure 1. Flow cytometric analysis of immunophenotypic markers expression in *hf*NPC cells

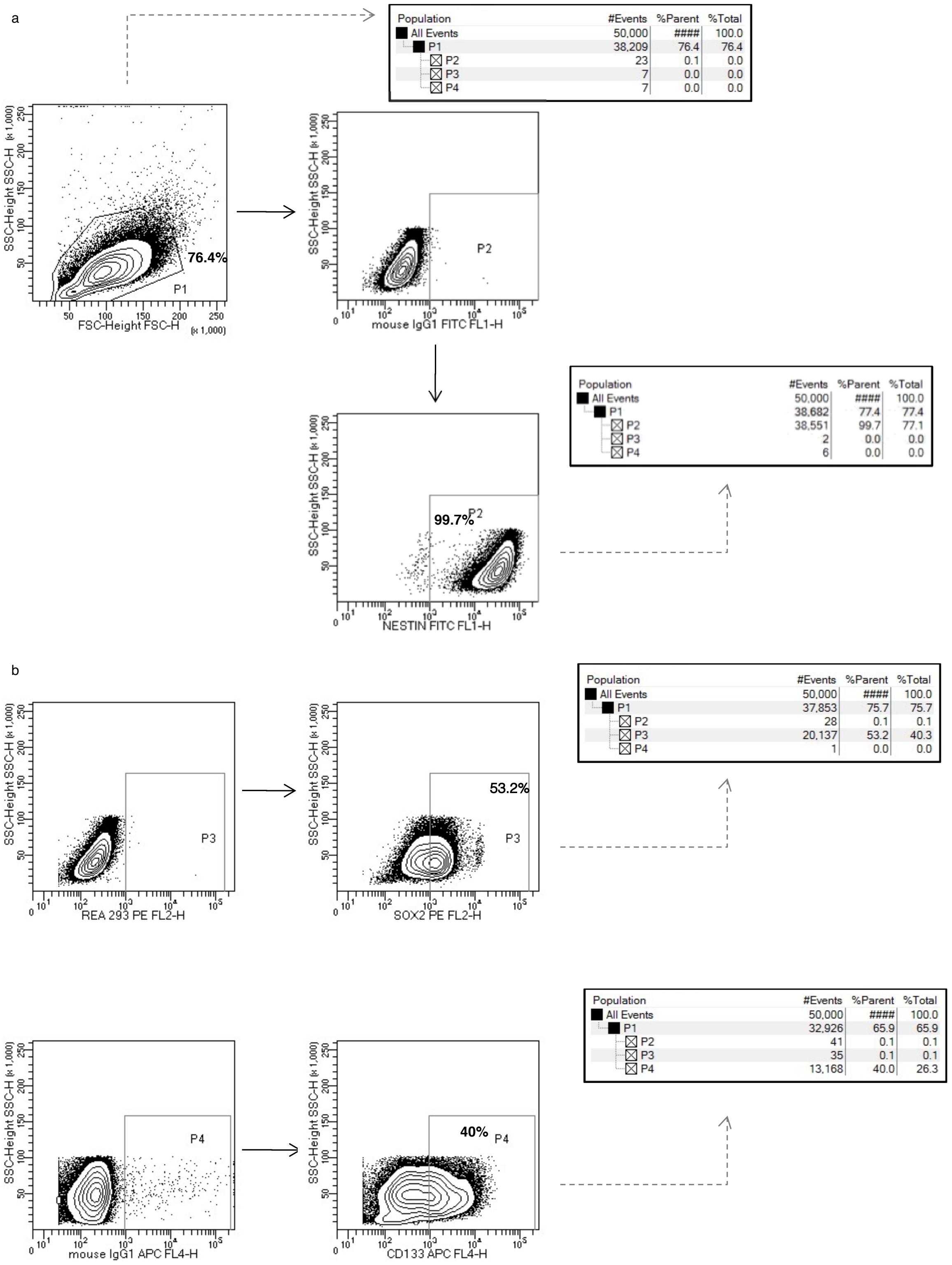

*(**a**) Dual contour plots illustrating the gating strategy adopted to the assess marker's expression is depicted for a representative case. Nucleated cells with low and intermediate physical scatter (FSC vs SSC) were selected and debris were excluded by gate P1 (top-left). Then P1 gated cells of the negative control sample were analyzed to establish the P2 marker set of positive vs negative fluorescence threshold (top-right) on a SSC vs Fluorescence dual contour plot. P2 marker set was then applied to the stained sample to calculate the % of positive cells (e.g. Nestin) according to the fluorescence channel (FITC, bottom-right). (**b**) The gating strategy was then applied to asses each marker's expression in different fluorescence channels. In the example P3 and P4 gates (both P1 gated) were used to assess the % of positive cells for SOX2 (top contour plots) and CD133 (bottom contour plots) respectively. Black arrows indicate the sequential steps of the gating strategy. Dotted arrows indicated the statistics box reporting both the number of cells and the % of positive cells for each relevant dual contour plots (with outliers). Each axis label indicates the marker and fluorochrome used.*

Supplementary Figure 2. Flow chart of the master cell bank production process

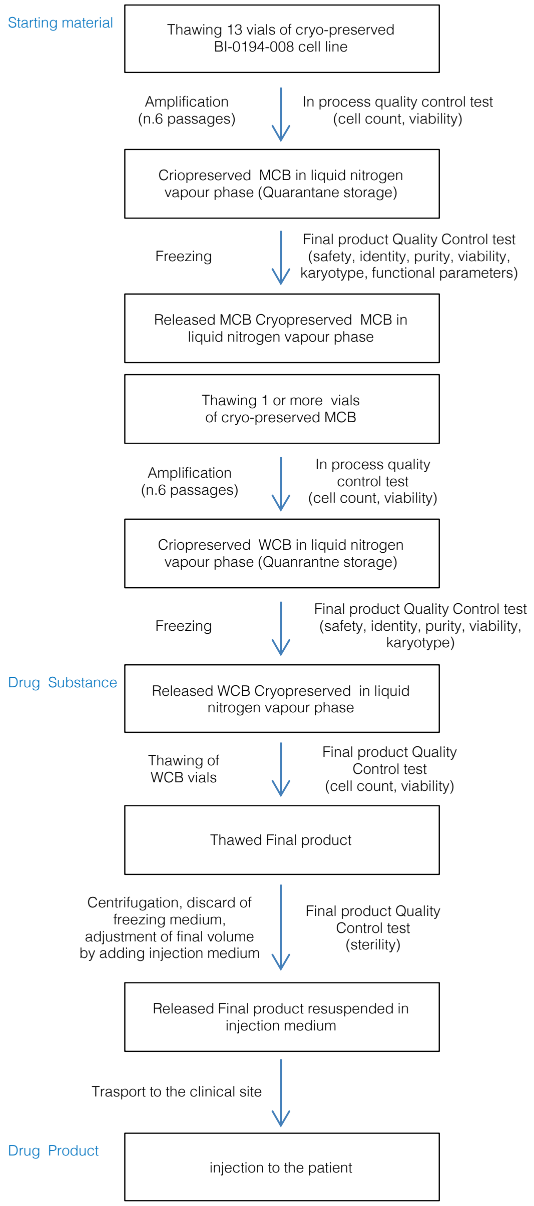

MCB: Master cell bank; WCB: Working cell bank.

Supplementary Figure 3. Microchimerism Analysis by droplet digital PCR

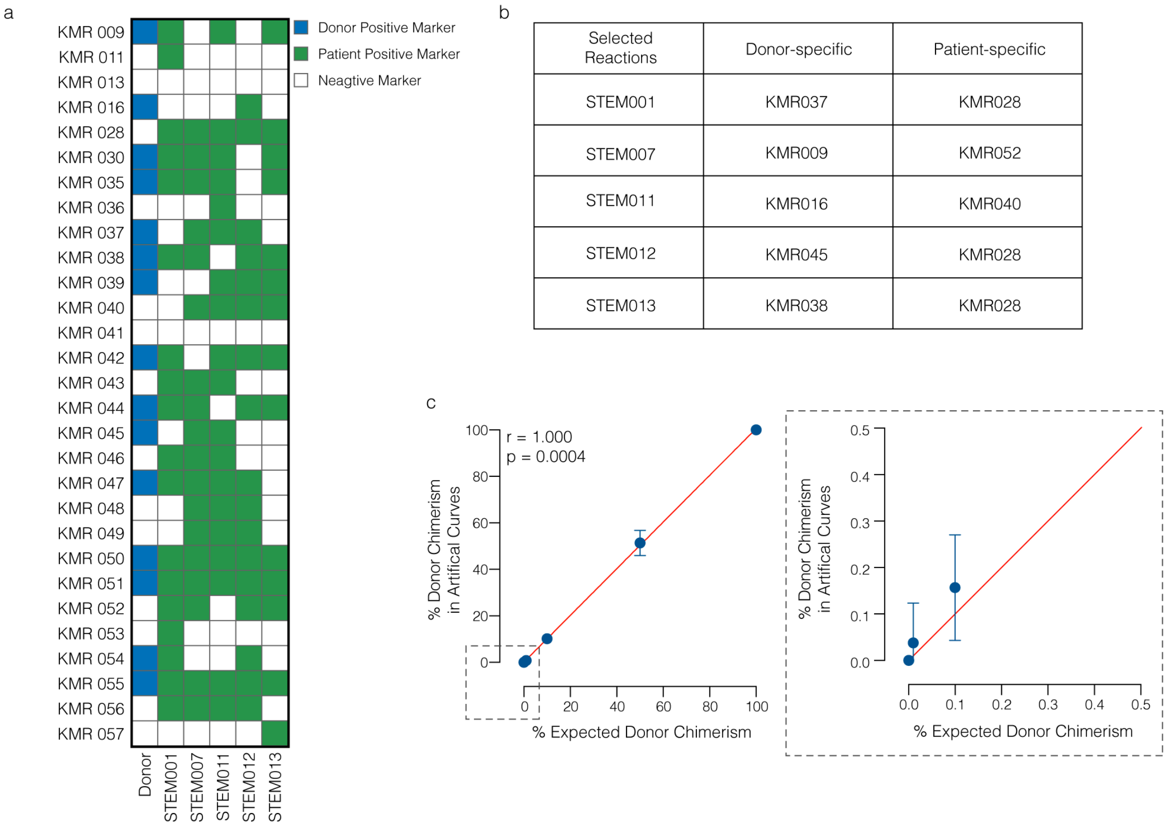

*(**A**) Screening results obtained by testing donor and patient genomic DNA extracted from PB samples using the commercial qPCR-based KMRtype genotyping assay. Colored squares represent genomic markers tested positive (blue) or negative (white) in the corresponding evaluated genomic DNA. (**B**) Markers selected to monitor donor- and patient-specific contributions to chimerism after treatment in each patient, based on the screening results. (**C**) Detection of donor chimerism in five artificial chimeric curves generated by mixing donor DNA with DNA from each of the #001, #007, #011, #012 and #013 patients. Dots indicate the average of chimerism measured from five independent artificial chimeric curves, and error bars the respective SDs. The red line indicates perfect correspondence between expected and observed results. R^2 and P value were calculated by a two-sided Spearman test at 95% confidence interval (CI).*

Supplementary Table 1. Plasma biomarkers changes

	Baseline	2-year Follow-up	P value
NfL pg/ml (mean± SD)	18.6 ± 8.9	35.5 ± 29.6	0.06
Tau pg/ml (mean± SD)	12.3 ± 14	21.4 ± 44.3	0.55
UCHL1 pg/ml (mean± SD)	51.4 ± 77.6	48 ± 50.6	0.77
GFAP pg/ml (mean± SD)	198.3± 80.2	267.8 ± 135.4	0.03

NfL: neurofilament light; UCHL1: ubiquitin C-terminal hydrolase-L1; GFAP: glial fibrillary acidic protein; SD: Standard Deviation; Two-sided Wilcoxon signed ranks test.

Supplementary Table 2. Longitudinal annual percentage changes of brain tissue volumes and spinal cord cross sectional area at two years

Mean annual volume changes % ± SD (W96 vs baseline)	All patients (All TCs)	*"Low dose"* (TC-A and B)	*"High dose"* (TC-C and D)	P value (Mann-Whitney U test)
PBVC	-0.90 ± 0.46	-1.12 ± 0.42	-0.69 ±0.42	0.09
PWMVC	-0.86 ± 0.40	-1.02 ± 0.29	-0.69 ± 0.44	0.13
PGMVC	-0.75 ± 0.33	-0.94 ± 0.23	-0.56 ± 0.31	0.04
PCSA	-1.39 ± 1.68	-1.79 ± 0.88	-0.89 ± 2.42	0.56
PnLTVC	-1.17± 1.48	-0.71 ± 1.06	-1.62 ± 1.78	0.31
PnRTVC	-0.77 ± 1.50	-0.74 ± 1.90	-0.8 ± 1.15	0.81

PBVC: percentage brain volume change, PWMVC: percentage white matter volume change, PGMVC: percentage grey matter volume change, PCSA: percentage cross sectional area change, PnLTVC: percentage normalized left thalamic volume change, PnRTVC: percentage normalized right thalamic volume change. SD: Standard deviation. Two-sided Mann-Whitney U test.

Supplementary Table 3. Experimental design of the *hf*NPC toxicity study in mice

Test Group	Treatment	Volume (uL)	Route	Number of Animals/Group	
				Males	Females
1	Untreated control	-	-	15	15
2	PBS + Cyclosporin	10	ICV	15	15
3	h-NPCs (about 10000000 cells/mouse) + Cyclosporin	10	ICV	15	15

PBS= Phosphate buffered saline; ICV=intracerebroventricular; h-NPCs= human neural precursor cells

Supplementary Table 4. Quality controls test

**Parameter**	**Specification**	**Results**	**Analytical method**
**Master Cell Bank (MCB)**			
Viability	>70.00%	71.78%	Trypan blue count (EP 2.7.29)
Barcode sequencing	Identity of the cell line	Homo sapiens	Sequencing of DNA using ABI 3500 Dx Genetic Analyzer
Sterility test	Sterile	Negative	EP 2.6.1
Mycoplasma	Negative	Negative	EP 2.6.7 corrected 6.1
AdventitiousViruses *in vitro*	Negative	Negative	28-days in vitro assay for the presence of viral contamination using 3 detector cell lines (Vero, MRC-5, and HeLa)
AdventitiousViruses *in vivo*	Negative	Negative	Mortality evaluation following test sample administration in suckling and adult mice and embryonated eggs

Virus-like (VLPs) and retrovirus-like (RVLPs) particles	Absence	Negative	Thin section transmission electron microscopy for cells
Human Virus	Negative	Negative	PCR Technique. Detection of HIV-1, HIV-2, HTLV-1, HTLV-2, EBV, CMV, HHV-6, HHV-7, HHV-8, HCV, HBV, SV40, PB19 and HAV sequences
Detection of bovine and porcine viruses	Negative	Negative	Assay according to 9 cfr for the following viruses: BAV1, BPV, BRSV, BVDV, PAV, PPV, Reo3, TGEV, Rabies, BTV, BPI3 and BHV-1/IBRV
DNA Fingerprinting	Positive	Pass (positive)	STR Fragment Analysis
Differentiation test	neurons (10-20%)<br>astrocytes (30-70%)<br>oligodendrocytes (0-2%)	Neurons 27%<br>Astrocytes 57%<br>Oligodendrocytes 1.8%	Immunofluorescence for the neuronal marker β-tubulin III, the astroglial marker GFAP and the oligodendroglial marker O4
LAL test (Endotoxin)	<0.50 EU/ml	≤0.48 EU/ml	EP 2.6.14
Karyotype	46, XY normal	46, XY normal	QFQ Banding
Immunophenotype	CD133 ≥ 10%<br>SOX2 ≥ 10%<br>NESTIN ≥ 10%	CD133=19.53%<br>SOX2=94.22%<br>NESTIN=98.88%	Flow Cytometry
**Working Cell Bank (WCB), n. 6 batches**			
Viability	>70.00%	mean 76.85% (range 70.12%-90.08%)	Trypan blue count (EP 2.7.29)
Sterility test	Sterile	Negative	EP 2.6.1
Mycoplasma	Negative	Negative	EP 2.6.7 corrected 6.1
LAL test (Endotoxin)	<0.50 EU/ml	≤0,50 EU/ml	EP 2.6.14
Karyotype	46, XY normal	46, XY normal*	QFQ Banding
Immunophenotype	CD133 ≥ 10%<br>SOX2 ≥ 10%<br>NESTIN ≥ 10%	mean CD133= 13.41% (range 11.46–15.95%); mean SOX2= 52.53% (range 18.65–78.69); mean NESTIN= 92.93% (range 89.91-96.60%)	Flow Cytometry
**Drug product (DC), n. 12 batches**			
Viability	>45.00%	mean 55.15% (range 45.23% - 66.50%)	Trypan blue count (EP 2.7.29)
Sterility test	Sterile	Negative	EP 2.6.1

**One batch could not be tested due to lack of metaphases*
