# Supplementary material for: Neural stem cell transplantation in patients with progressive multiple sclerosis: an open-label, phase 1 study
Source: Nat Med. 2023 Jan 9;29(1):75–85. doi: 10.1038/s41591-022-02097-3 (PMC9873560; doi:10.1038/s41591-022-02097-3)
Supplement: Supplementary file 2 — Reporting Summary [file 41591_2022_2097_MOESM2_ESM.pdf]

nature research

Corresponding author(s): Gianvito Martino

Last updated by author(s): Oct 10, 2022

Reporting Summary

Nature Research wishes to improve the reproducibility of the work that we publish. This form provides structure for consistency and transparency in reporting. For further information on Nature Research policies, see our Editorial Policies and the Editorial Policy Checklist.

Statistics

For all statistical analyses, confirm that the following items are present in the figure legend, table legend, main text, or Methods section.

n/a	Confirmed	
☐	☒	The exact sample size (*n*) for each experimental group/condition, given as a discrete number and unit of measurement
☐	☒	A statement on whether measurements were taken from distinct samples or whether the same sample was measured repeatedly
☐	☒	The statistical test(s) used AND whether they are one- or two-sided<br>*Only common tests should be described solely by name; describe more complex techniques in the Methods section.*
☐	☒	A description of all covariates tested
☐	☒	A description of any assumptions or corrections, such as tests of normality and adjustment for multiple comparisons
☐	☒	A full description of the statistical parameters including central tendency (e.g. means) or other basic estimates (e.g. regression coefficient) AND variation (e.g. standard deviation) or associated estimates of uncertainty (e.g. confidence intervals)
☐	☒	For null hypothesis testing, the test statistic (e.g. *F*, *t*, *r*) with confidence intervals, effect sizes, degrees of freedom and *P* value noted<br>*Give P values as exact values whenever suitable.*
☒	☐	For Bayesian analysis, information on the choice of priors and Markov chain Monte Carlo settings
☒	☐	For hierarchical and complex designs, identification of the appropriate level for tests and full reporting of outcomes
☒	☐	Estimates of effect sizes (e.g. Cohen's *d*, Pearson's *r*), indicating how they were calculated

Our web collection on statistics for biologists contains articles on many of the points above.

Software and code

Policy information about availability of computer code

Data collection

BD FACSCalibur™ (BD Biosciences)
Leica TCS SP5 Laser Scanning Confocal (Leica, Wetzlar, Germany)
Cadwell MS10 magnetic stimulator
Spectral domain Spectralis OCT (Heidelberg Engineering, Heidelberg, Germany; software v5.8)
1.5 Tesla MR scanner system (Achieva dStream; Philips Medical Systems, Best, The Netherlands)
HD-1 Simoa (Quanterix, Lexington, MA, USA)
Nanodrop spectrophotometer (Thermo Scientific, Franklin, MA, USA)
QX200 Droplet Reader (Bio-Rad Laboratories, Hercules, CA, USA)
Luminex100
Q-Exactive mass spectrometer (Thermo Scientific, Bremen, Germany) with a nano-electrospray ion source (Proxeon Biosystems) and a nUPLC Easy nLC 1000 (Proxeon Biosystems)

Data analysis

FACSDiva and BD CellQuest™ Pro (BD Biosciences)
nicMSlesions, relase 0.2, python version 2.7
SIENAX version 2.6, FSL 5.0.1
Statistical Parametric Mapping (SPM) toolbox version 12; Matlab version 8.0 (Release 2012b)
FSL 5.0.1 FIRST software
Jim software package (Version 7, Xinapse Systems, Colchester, UK)
QuantaSoft™ software (Bio-Rad Laboratories, Hercules, CA, USA)
Bio-plex manager v.6.0
Mascot (version 2.6, Matrix Science)
MaxQuant1 software (version 1.6.1.0)
MasterView v 1.1

MetaboAnalyst v. 4.0
MeV software v. 4_9_0
Mass Metabolite Spectral Library (SCIEX)
Gprofiler2 R library (v.0.2.1, R version 3.6.3)
Morpheus analysis software (https://software.broadinstitute.org/morpheus/)
SPSS statistical package (24.0 Version)
GraphPad Prism (version 8.0, GraphPad Software, San Diego, CA)
prcomp R package (version 3.5.1)
GSEA algorithm, version 4.1.015

For manuscripts utilizing custom algorithms or software that are central to the research but not yet described in published literature, software must be made available to editors and reviewers. We strongly encourage code deposition in a community repository (e.g. GitHub). See the Nature Research guidelines for submitting code & software for further information.

Data

Policy information about availability of data

All manuscripts must include a data availability statement. This statement should provide the following information, where applicable:

- Accession codes, unique identifiers, or web links for publicly available datasets
- A list of figures that have associated raw data
- A description of any restrictions on data availability

Pseudonymized participant data including baseline characteristics and results of primary, and exploratory endpoint analyses reported in this article can be shared in compliance with current data protection regulations by the European Union. Data sharing requires a current and positive vote by the requestor's competent ethics committee. All proposals should be directed to the corresponding author. The mass spectrometry proteomics data have been deposited to the ProteomeXchange Consortium via the PRIDE partner repository with the datasets identifier (i) PXD034840 and 10.6019/PXD034840 for the low dose experiment, and (ii) PXD034846 and 10.6019/PXD034846 for the high dose experiment.

Field-specific reporting

Please select the one below that is the best fit for your research. If you are not sure, read the appropriate sections before making your selection.

☒ Life sciences ☐ Behavioural & social sciences ☐ Ecological, evolutionary & environmental sciences

For a reference copy of the document with all sections, see nature.com/documents/nr-reporting-summary-flat.pdf

Life sciences study design

All studies must disclose on these points even when the disclosure is negative.

Sample size

This study was a phase I clinical trial evaluating the safety of human fetal neural precursor cells (hfNPCs) in humans with multiple sclerosis for the first time. Based on this premise a formal sample size calculation cannot be performed. We planned to test a single escalating dose of hfNPCs in four different treatment cohorts (TCs). Each TC consisted of three patients at minimum, and it would be increased to six patients in case of safety issue. We used the standard 3 + 3 design, which remain one of the most utilized designs due to simplicity of execution and robustness in phase I clinical trials.

Data exclusions

No data were excluded from the analyses.

Replication

This is a prospectively, first in patients with MS, phase I clinical trial. Due to the limited sample size and available samples, the experiments described in the Results were not replicated. Replication of clinical and related translational experiments will require a new clinical trial.

Randomization

Each patient was consecutively enrolled each TC for safety reasons. After the inclusion of the first patient in each TC, we waited at least 14 days before beginning the treatment of the subsequent patient and maintained the same interval for all patients within the same TC.
Once all patients of the first TC had been treated, if no dose limiting toxicities (DLTs) were reported, we waited 3 months before starting the administration in patients within the following TC dose. If 1 DLT occurred within the TC, the cohort would have been extended to 6 patients. Instead, if more than 1 DLT occurred, the current dosage would have been considered excessive, and the immediate lower dosage would have been considered the maximum tolerated dose (MTD).

Blinding

This trial was open label with no blinding.

Reporting for specific materials, systems and methods

We require information from authors about some types of materials, experimental systems and methods used in many studies. Here, indicate whether each material, system or method listed is relevant to your study. If you are not sure if a list item applies to your research, read the appropriate section before selecting a response.

Materials & experimental systems

n/a	Involved in the study
☐	☒ Antibodies
☐	☒ Eukaryotic cell lines
☒	☐ Palaeontology and archaeology
☐	☒ Animals and other organisms
☐	☒ Human research participants
☐	☒ Clinical data
☒	☐ Dual use research of concern

Methods

n/a	Involved in the study
☒	☐ ChIP-seq
☐	☒ Flow cytometry
☐	☒ MRI-based neuroimaging

Antibodies

Antibodies used

cod. IC1259F Human Nestin Fluorescein-conjugated Antibody R&D SYSTEMS
cod. 130-098-829 CD133/1 (AC133)-APC, human 30 tests Miltenyi Biotech
cod. 130-104-994 Anti-Sox2-PE, h&m 30 tests Miltenyi Biotech
cod. 21330443 anti-Human CD44 FITC Immunotools
cod 555974 PE anti-human CD184(CXCR4) BD Bioscience
cod. 130-123-935 CD29 Antibody, anti-human, FITC Miltenyi Biotech
cod. Z0334 Rabbit Anti-Glial Fibrillary Acidic Protein (GFAP) Antibody ,Dako
cod. 802001 Rabbit Anti-β TUBOLIN III clone TUJ1 BIOLEGEND
cod. O7139 mouse ANTI-OLIGODENDROCYTE MARKER O4, CLONE O4 Sigma-Aldrich
Cat # A-21206 Donkey anti-Rabbit IgG (H+L) Highly Cross-Adsorbed Secondary Antibody, Alexa Fluor™ 488 Invitrogen
Cat # A-21042 Goat anti-Mouse IgM (Heavy chain) Cross-Adsorbed Secondary Antibody, Alexa Fluor™ 488 Invitrogen

Validation

All antibodies used in our study are commercially available and validated by manufacturer. https://www.rndsystems.com/datasheet/coa; https://www.miltenyibiotec.com/IT-en/products/macs-antibodies/antibody-validation.html?countryRedirected=1#gref; https://www.bdbiosciences.com/en-us/products/reagents/flow-cytometry-reagents; https://www.agilent.com/en/product/dako-omnis-solution-for-ihc-ish/primary-antibodies-for-dako-omnis; https://www.biolegend.com/en-us/quality/quality-control; https://www.merckmillipore.com/IT/it/life-science-research/antibodies-assays/antibodies-overview/Antibody-Development-and-Validation/cFOb.qB.8McAAAFOb64qQvSS,nav; https://www.thermofisher.com/ch/en/home/life-science/antibodies/invitrogen-antibody-validation.html.

Eukaryotic cell lines

Policy information about cell lines

Cell line source(s)

Non-immortalized hfNPCs cell line (#BI-0194-008) was obtained from the telencephalon and diencephalon of a single 10/12-week post-conception (WPC) human fetus, after elective pregnancy interruption, provided by Fondazione Ca' Granda (Milan).

Authentication

Identity of the cell line (homo sapiens) was confirmed by barcode DNA sequencing using ABI 3500 Dx Genetic Analyzer; Karyotype (46, xy) by QFQ Banding method; DNA Fingerprinting by STR Fragment Analysis.

Mycoplasma contamination

We demonstrated the adsence of mycoplasma in #BI-0194-008 cell line.

Commonly misidentified lines
(See ICLAC register)

No commonly misidentified cell lines were used

Animals and other organisms

Policy information about studies involving animals; ARRIVE guidelines recommended for reporting animal research

Laboratory animals

CD1 mouse, Charles River Italia. Age: 6 weeks. Male and Female. The animals were housed in Individual Ventilated Cages. The mice were kept in 12hour/12hour light/dark cycles, at 21.5°C +/- 1.5°C temperature and 55% +/- 15 humidity. Mice were fed with gamma irradiated food and water.

Wild animals

The study did not invole wild animals.

Field-collected samples

The study did not involve samples collected from the field.

Ethics oversight

This study was conducted by Accelera s.r.l. in compliance with:
Italian Legislative Decree (D.L. No. 50 dated March 2 , 2007) as published in G.U. No. 86 of April 13th, 2007;
Organisation for Economic Co-operation and Development (OECD) Principles of Good Laboratory Practice (GLP) [C(97) 186 (Final)].
The Principles of Good Laboratory Practice are accepted by the Regulatory Authorities of United States of America and Japan on the basis of intergovernmental agreements.

Note that full information on the approval of the study protocol must also be provided in the manuscript.

Human research participants

Policy information about studies involving human research participants

Population characteristics	Recruited patients were required to meet the following inclusion criteria: signature of the Informed Consent Form by the patient or patients' legal tutors; age 18 to 55 years; diagnosis of PMS as per the revised MC Donald 2010 criteria with a progressive course according to 2013 Lublin phenotypes classification with failure or intolerance to all approved therapies according to the disease course or without any alternative approved therapy; disease duration 2 to 20 years; EDSS ≥ 6.5; evidence of progression of the disease defined by an increase of ≥ 0.5 EDSS points in the last 12 months; presence of oligoclonal bands (OBs) in the CSF required for PPMS.
Recruitment	Patients were all recruited from our center by neurologist who daily follow people with progressive multiple sclerosis. Patients who met eligibility criteria were enrolled in the order in which they contacted our program for potential inclusion in the study. All patients who met eligibility criteria were offered the trial. Partecipants were not compesated, except for the reimbursement of reasonable travel expenses. It is not considered there was any selection bias in recruitment of patients into the trial.
Ethics oversight	The study was conducted in compliance with the Declaration of Helsinki and Good Clinical Practice. It was approved by the San Raffaele Scientific Institute (Milan, Italy) ethics committee and was authorized by the AIFA (Italian Medicines Agency). All participants provided written consent to participate. None of the study participants received compensation for participation in the study. The trial is registered at ClinicalTrials.gov (NCT0326907) and the European Union Clinical Trials Register (EudraCT no. 2016-002020-86).

Note that full information on the approval of the study protocol must also be provided in the manuscript.

Clinical data

Policy information about clinical studies

All manuscripts should comply with the ICMJE guidelines for publication of clinical research and a completed CONSORT checklist must be included with all submissions.

Clinical trial registration	NCT03269071; EudraCT no. 2016-002020-86
Study protocol	The study protocol is available upon reasonable request.
Data collection	The safety data were collecter from the enrollment up to 2 years after the hfNPCs transplantation during the visits at the department of Neurolofy, San Raffaele Hospital, Italy (From May 2017 to June 2021). CSF samples were collected at baseline and 3 months after the hfNPCs transplantation. Brain and spinal cord MRI scans were acquired at baseline and at 3, 6, 12, and 24 months after the transplantation. Plasma samples were collected immediately before and 1, 3, 6, 12, and 24 months after transplantation. The last patient last visit was performed on June 2021.
Outcomes	The primary objective of the study is to evaluate the feasibility, safety and tolerability of intrathecally administered hfNPCs in patients affected by progressive multiple sclerosis. As usual in a phase I study. The documented AE severity was classified according the Common Terminology Criteria for Adverse Events (CTCAE) (Grade 1= mild; Grade 2= moderate; Grade 3= severe; Grade 4= life-threatening; Grade 5: death), and the attribution to the investigational medicinal product (unrelated, unlikely, possibly, probably, definitely) was defined accordingly to NCI guidelines of AE reporting requirements. Serious adverse event (SAE) was defined as any untoward expected medical occurrence or effect that at any dose results in death, that is life-threatening, that requires hospitalization or prolongation of existing hospitalization, that results in persistent or significant disability or incapacity, or that is a congenital anomaly or birth defect. The safety profile of hfNPCs was evaluated with 22 follow up visits, over a period of 96 weeks after administration, monitoring for survival, safety, tolerability, and overall changes in the neurological status. Patients have been evaluated by a complete physical examination, clinical laboratory tests, and instrumental follow-up (electrocardiogram and chest x-ray at 1 and 2 years, abdominal ultrasound at 2 years). A diagnostic lumbar puncture was performed for safety reasons 3 months after the hfNPC injection in all the treated patients to rule out the presence of infective or aseptic meningitis or obstructive hydrocephalus. We evaluated the AEs prevalence and severity in each treatment cohort.

Flow Cytometry

Plots

Confirm that:

☒ The axis labels state the marker and fluorochrome used (e.g. CD4-FITC).

☒ The axis scales are clearly visible. Include numbers along axes only for bottom left plot of group (a 'group' is an analysis of identical markers).

☒ All plots are contour plots with outliers or pseudocolor plots.

☒ A numerical value for number of cells or percentage (with statistics) is provided.

Methodology

Sample preparation	Surface staining: Cultured cells were diluted 1:2 with PBS. Centrifuged at 300g for 5 min. at room temperature (RT). Pellet were resuspended at 500,000 cells/100 uL . Then 20 ul of FcR Blocking Reagent was added to each tube. Incubate for 10 min. in the dark at RT. Then add the monoclonal antibody and incubate for 10 min. at +4°C. Wash with 2mL of PBS and resuspend

	in 400 uL of PBS and proceed with FACS acquisition.<br>Cytoplasmic staining: Cultured cells were diluted 1:2 with PBS. Centrifuged at 300g for 5 min. at RT. Pellet were resuspended at 500,000 cells/100 uL. Add 1 mL/tube of Fix/Perm solution (FoxP3 staining Buffer set, Miltenyi Biotec) and incubate for 30 min at +4°C in the dark. Wash 1x with PBS, centrifuge at 300g for 5 min. at RT. Wash 1x with cold Perm Buffer (FoxP3 staining Buffer set, Miltenyi Biotec) and resuspend the cells in 100 ul of Perm Buffer. Add monoclonal antibody and incubate for 30 min. in the dark. Wash the cells with Perm Buffer, centrifuge at 300g for 5 min. at RT , resuspend in 400 uL of PBS and proceed with FACS acquisition.
Instrument	BD FACSCalibur™ (BD Biosciences)
Software	FACSDiva and BD CellQuest™ Pro (BD Biosciences)
Cell population abundance	Mean abundance of CD133+, Nestin+, and SOX2+: 11.66%, 92.87%, and 52.47% respectively.
Gating strategy	Gate nucleated cells on FSC/SSC dual dot plot excluding debris. Set markers threshold for each fluorescence (e.g. FITC) on SSC/Fluorescence dual dot plot, using the negative control tube. Calculate the % of positive cells for each immunophenotypic marker tested (e.g CD133) on SSC/Fluorescence dual dot plot.

☒ Tick this box to confirm that a figure exemplifying the gating strategy is provided in the Supplementary Information.

Magnetic resonance imaging

Experimental design

Design type	not applicable
Design specifications	not applicable
Behavioral performance measures	not applicable

Acquisition

Imaging type(s)	Structural
Field strength	1.5 T
Sequence & imaging parameters	I. Brain sequences: FOV [mm2], matrix, Slice Thickness (ST) [mm], orientation, Repetition Time (TR) [mm]/Echo Time (TE) [mm]/Flip Angle (FA) [degree]<br>a) 3D FLAIR, Turbo Spin Echo (TSE): FOV=230x230, matrix=200x200, ST=1.15, sagittal, TR/TE/FA=4800/277/90°;<br>b) T2-weighted TSE: FOV=230x192, matrix=384x224, ST=5, axial, TR/TE/FA=4446/100/90°;<br>c) T2-weighted fast field echo (FFE): FOV=256x256, matrix=256x204, ST=5, axial, TR/TE/FA=698/23/13º;<br>d) Diffusion weighted EPI: FOV=240x240, matrix=116x94, ST=4, axial, TR/TE/FA=4043/65/90º;<br>e) 3D T1-weighted turbo field echo (TFE):, FOV=256x256, matrix=256x256, ST=1.2, sagittal, TR/TE/FA=8/3.7/8º;<br>f) Postcontrast 2D T1-weighted TSE: FOV=230x183, matrix= 272x164, ST=5, axial, TR/TE/FA=580/12/69º;<br>g) postcontrast 3D T1-weighted TFE: same parameters as for sequence e).<br><br>II. Spinal cord sequences:<br>a) cervical and dorsal 2D STIR: FOV=380x380, matrix=424x296, ST=3, sagittal, TR/TE/FA=2500/50/90°;<br>b) cervical cord 3D T1-weighted TFE: FOV=250x250, matrix=256x256, ST=1, sagittal, TR/TE/FA=8.2/3.8/8º;<br>c) cervical and dorsal 2D T1-weighted TSE:, FOV=380x380, matrix=380x304, ST=3 mm, sagittal, TR/TE/FA=400/7.4/90°);<br>d) cervical and dorsal 2D T2-weighted TSE: FOV=380x380, matrix: 424x300, ST=3, sagittal, TR/TE/FA=3748/120/90°;<br>e) post contrast cervical and dorsal 2D T1-weighted TSE, with the same parameters as for sequence c).
Area of acquisition	Whole brain, cervical and dorsal spinal cord

Diffusion MRI ☒ Used ☐ Not used

Parameters: Single shell, b-value= 1000, 3 DW directions, no cardiac gated

Preprocessing

Preprocessing software	Lesion segmentation: nicMSlesions, Release 0.2, 2019<br>Whole brain atrophy: FSL (version 5.0.5), SIENAx software<br>Percentage Brain volume change: FSL (version 5.0.5), SIENA software<br>Percentage of GM and WM volume changes: MATLAB (Release 2012 - 8.0), SPM12 Toolbox<br>Thalami segmentation: FSL (version 5.0.5), FIRST software<br>Cervical cord area: Jim software package (Version 7, Xinapse Systems, Colchester, UK)
Normalization	None
Normalization template	None

Noise and artifact removal | The presence of artifacts was assessed by visual inspection

Volume censoring | Not applicable

Statistical modeling & inference

Model type and settings | None

Effect(s) tested | None

Specify type of analysis: ☐ Whole brain ☐ ROI-based ☐ Both

Statistic type for inference (See Eklund et al. 2016) | None

Correction | Not applicable

Models & analysis

n/a	Involved in the study
☒	☐ Functional and/or effective connectivity
☒	☐ Graph analysis
☐	☒ Multivariate modeling or predictive analysis

Multivariate modeling and predictive analysis | A multivariate regression analysis was run to evaluate the relationship between the number and volume of new brain T2 lesions, the number of GELs and the regional brain volumes with the number of infused cells, after adjusting for baseline volumes (whole brain and GM), age, EDSS and for T2 lesion volume change at two years.
